# Anti colorectal cancer activity and in silico studies of novel pyridine nortopsentin analog as cyclin dependent kinase 6 inhibitor

**DOI:** 10.1038/s41598-024-75411-3

**Published:** 2024-11-01

**Authors:** Heba Abdelmegeed, Heba M. Abo-Salem, Ehab M. Zayed, Eslam R. El-Sawy

**Affiliations:** 1 Chemistry of Natural Compounds Department, Pharmaceutical and Drug Industries Research Institute, National Research Centre, Dokki, 12622 Giza Egypt; 2 Green Chemistry Department, National Research Centre, Dokki, 12622 Giza Egypt

**Keywords:** Cancer, Chemical biology, Drug discovery, Chemistry

## Abstract

Nortopsentins are a vital class of deep-sea sponge metabolites which can be used as leads for antitumor agents. Although their action has been studied in several diseases’ contexts, their cytotoxic activity against colorectal carcinoma has not yet been fully investigated. Therefore, a series of 2,6-bis(1*H*-indol-3-yl)-4-(substituted-phenyl)pyridin-5-carbonitriles **4a–j** (nortopsentin analogs) was investigated for their cytotoxic activity against colorectal carcinoma. The analog **4i** showed the highest antitumor activity via inducing cell cycle arrest at G1 phase. Cell cycle arrest was induced due to expression downregulation of CDK2, CDK4, and CDK6. In addition, **4i** suppressed the enzymatic activity of CDK6. The theoretical study of some basic quantum factors and the geometric shape of compound **4i** proved that the compound is stable and a soft molecule, in which the E_HOMO_ and E_LUMO_ energies were negative and had a small ∆E gap. **4i** also demonstrated a high potential for oral bioavailability due to its adherence to Lipinski’s rule of five. The molecular docking studies of **4i** analog showed good binding mode with CDK6 active pocket through the formation of multiple interactions with its key amino acids.

**Supplementary Information:**

The online version contains supplementary material available at 10.1038/s41598-024-75411-3.

## Introduction

Colorectal cancer is the third most common cancer type which is responsible for an enormous number of deaths reaching 881,000 deaths worldwide in 2018^1,2^. Advancements in diagnostic measures led to its early detection, hence early treatment. The main treatment for colorectal cancer is endoscopic and surgical excision in addition to preoperative radiotherapy which are extremely invasive compared with small-molecule targeting therapy^1^. Since, the molecular basis for colorectal cancer development and its underlying causes have largely been identified^3,4^, the discovery of novel small molecules against colorectal cancer is inevitable. One of the main mechanisms for cancer development is cell cycle dysregulation. Cell cycle is a well-controlled process that requires multiple checkpoints in order to prevent uncontrolled cell division. Each phase of the cell cycle is regulated by a complex network of mechanisms to ensure the absence of any errors before they proceed to the following phase. Evading these controls leads to DNA damage accumulation and continuous cell division giving rise to cancerous cells^5^. Cyclin-dependent kinases (CDKs) are a family of enzymes that control cell cycle through interacting with different cyclins. Changes in CDKs activity regulate cell cycle entry, progression, and cell division^6^. Various cyclins interact with CDKs forming stable complexes leading to CDKs phosphorylation and activation^7^. Cyclin–CDK complexes in human network are composed of more than 20 CDKs and up to 30 distinct cyclin proteins^8^. The activation of these complexes is induced by several mitogenic signals and inhibited by cell-cycle checkpoints^6^. Therefore, CDKs are substantially highly expressed in cancer tissues compared with normal tissues and their dysregulation is closely associated with tumorigenesis^9^. The classical cell cycle CDKs are Cdk1, Cdk2, Cdk4, and Cdk6 which coordinate cell cycle progression. For the cell cycle to start, mitogenic signals induce the expression of the D-type cyclins that preferentially bind and activate both CDK4 and CDK6 leading to G1/S phase transition^10–12^. This activation of cyclinD-CDK4/6 complexes upregulates E-type cyclins which in turn bind to CKD2 forming a complex that is essential to further drive the transition from G1 triggering S phase^13^. CDK1 is another cyclin dependent kinase which is essential for cell cycle progression where it promotes the G2/M phase transition by binding to cyclin A, leading to mitotic entry^14,15^. Hence, CDKs have become attractive targets against different types of cancer such as breast cancer^7^ and solid tumors^16^. A wide variety of CDK inhibitors have been developed to suppress cell cycle progression^17^. For instance, inhibiting CDK4/6 hyperactivity using small molecules led to different potent antiproliferative agents against retinoblastoma (Rb)-positive tumor cells. These CDK4/6 inhibitors induced an exclusive G1 arrest, such as palbociclib, abemaciclib, ribociclib, and Trilaciclib^11^. Hence, developing inhibitors targeting CDK4/6 is considered a promising strategy for suppressing tumorigenesis.

**Fig. 1:**
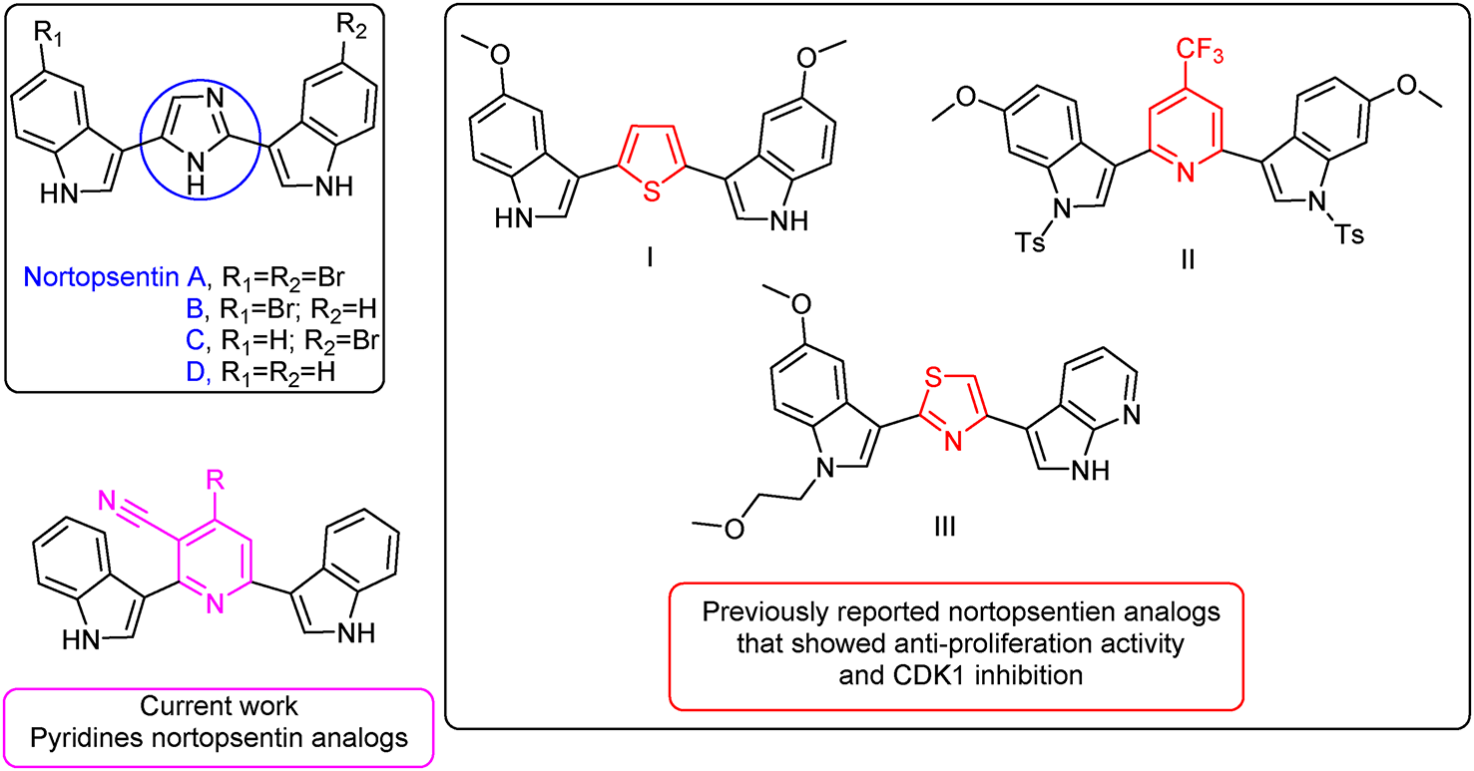
Chemical structures of natural nortopsentin A–D and its analogs.

The marine environment is a superb source of natural bioactive compounds, especially anti-tumor agents^18,19^. Nortopsentins A–D represents an important class of deep-sea sponge metabolites, useful as leads for antitumor agents^20^. Nortopsentins A–D (Fig. 1) are characterized by two indole units linked, through their position 3, by an imidazole core as a spacer which had been isolated from *Spongosorites ruetzleri*^20^. Several nortopsentin analogs have been designed where different heterocyclic rings replaced the imidazole ring of the natural compound. These analogs showed anti-proliferative activity^21–23^ where some of which inhibited CDK1^24–26^. Figure 1 displays some examples of these analogs. The thiophene nortopsentin analog (I) was particularly effective against the leukemia subpanel (CCRF-CEM (GI50, MOLT-4, HL60 TB), and RPMI-8226) with GI_50_ values ranging from 0.34 to 3.54 μM^21^. On the other hand, the pyridine nortopsentin analog (bis(indolyl)-4-trifluoromethylpyridine, II) showed significant cytotoxic activity with IC_50_ values of 4.3 and 1.7 μM against P388 and A549 cell lines, respectively^27^. For CDK1, the thiazole nortopsentin analog (III) demonstrated antitumor activity against MCF7 cancer cell line via inhibiting CDK1 activity in vitro where the viable cells were trapped in G2/M phase^24^.

Based on these findings, our present work aims to utilize the pyridine nortopsentin analogs we previously prepared^28^ to investigate their antitumor activity against several cancer cell lines. Next, we examined the anti-proliferative mechanism against the cancer cell lines affected by the most active analog. Moreover, we conducted an in-silico study including an analysis of certain basic quantum parameters, an analysis of the geometric shape, and drug-likeness (ADME). Finally, a molecular docking study was performed to elucidate the potential binding patterns of the active nortopsentin analog against the active pocket of CDK6.

## Results

### Chemistry

**Fig. 2:**
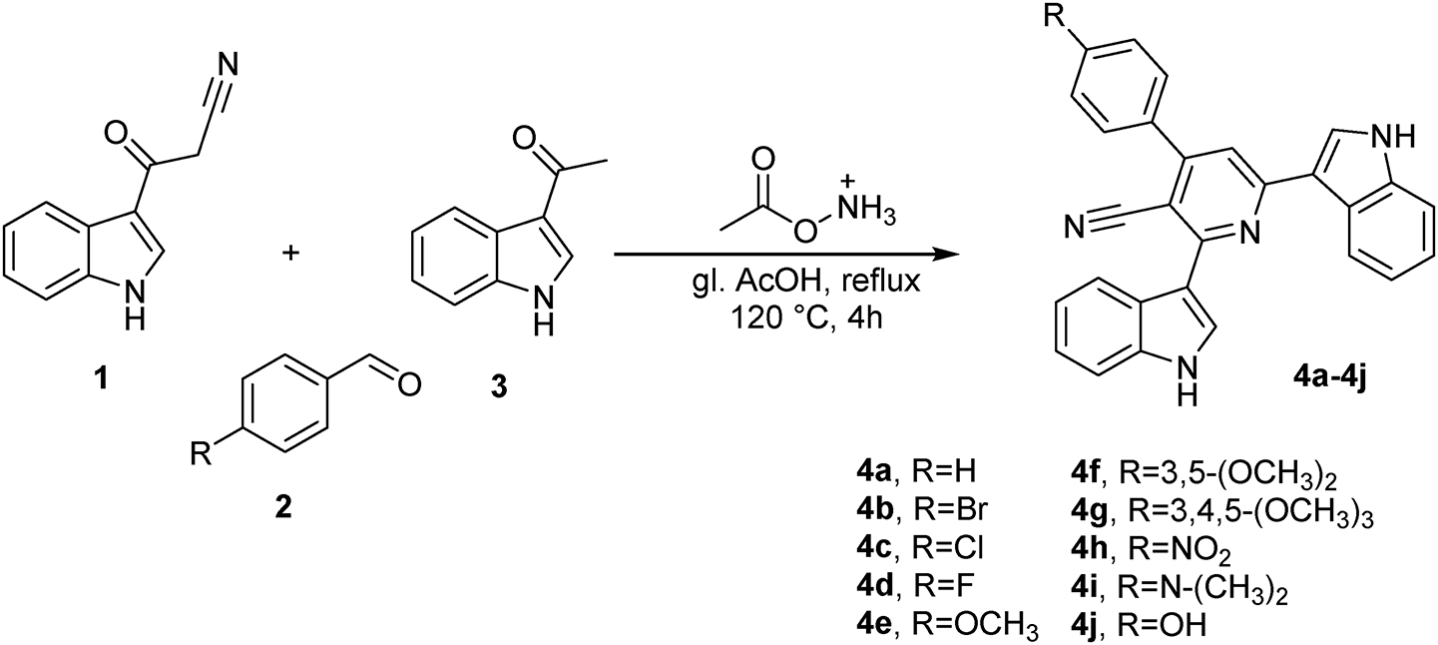
Pyridine nortopsentin analogs **(4a–j)**.

Figure 2 illustrates the chemical pathway of our previous work for obtaining pyridine nortopsentin analogs (2,6-bis(1*H*-indol-3-yl)-4-(substituted-phenyl)pyridin-5-carbonitriles **(4a–j)**^28^. Hence, we investigated their antitumor activity against several cancer cell lines.

### Nortopsentin analog 4i exhibited cytotoxic activity against human colorectal carcinoma

**Fig. 3:**
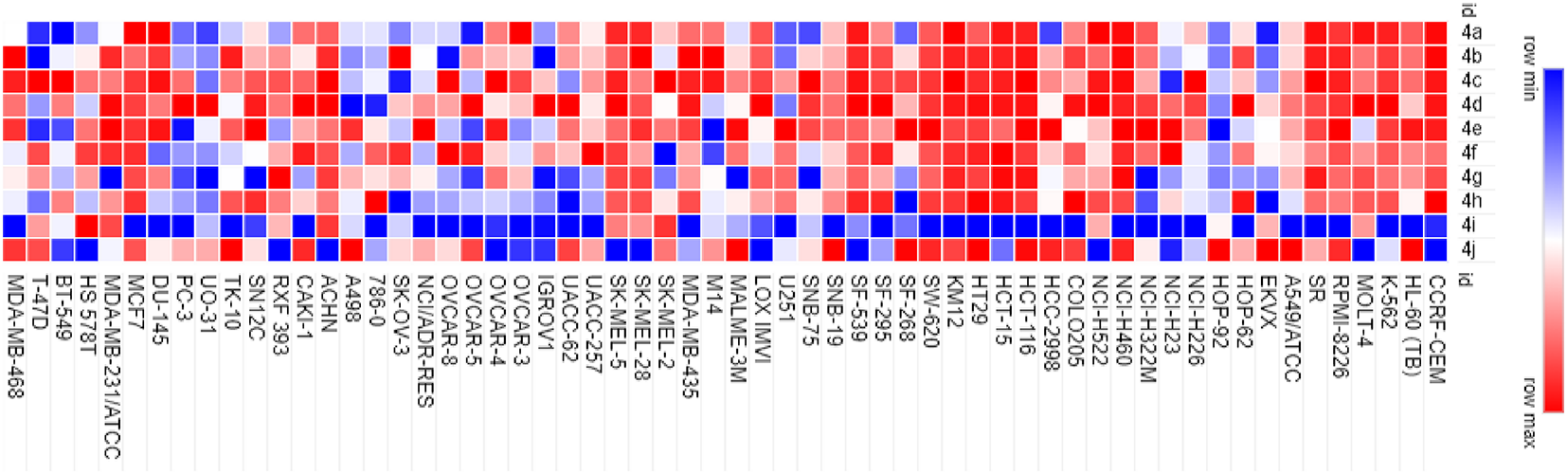
Heatmap of nortopsentin analogs (**4a–4j**) demonstrating their effect on colorectal cancer cells viability.

In order to determine the cytotoxic activity of nortopsentin analogs (**4a**–**4j**), their antitumor activity was measured against 60 different cell lines (Supplementary Table s1). The heatmap in Fig. 3 demonstrates the antitumor activity of all nortopsentin analogs against all tested cancer cell lines where the red color indicates higher tumor cells viability while the blue color indicates less tumor cells viability. The analog **4i** exhibited the highest level of cytotoxic activity against almost all tumor cell lines. Colorectal cancer was one of the tumors against which **4i** analog showed the highest cytotoxic activity, hence we focused on investigating **4i** cytotoxic mechanism of action. Using the MTT assay test, the analog **4i** showed IC_50_ in the nanomolar range (28.8 nM) compared with doxorubicin that showed IC_50_ of 10.11 µM. This demonstrates that **4i** analog is a very potent cytotoxic compound against colorectal cancer.

### Nortopsentin 4i analog induced cell cycle arrest at G1-phase

**Fig. 4:**
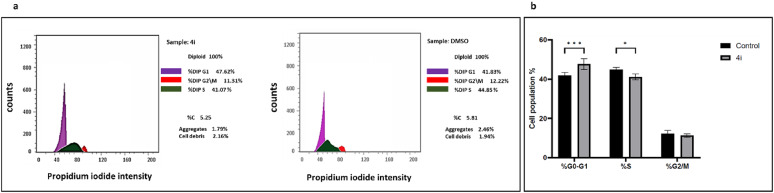
(**a**) Histograms of cell cycle phases using PI staining for FACS analysis. (**b**) Percentages of cells accumulation in all cell cycle phases induced by **4i** analog versus control cells. Data are represented as the mean ± SD of three independent experiments (Fig. s1). Statistical analysis was conducted using one-way ANOVA followed by Bonferroni’s multiple comparison test; *p < 0.05, **p < 0.01, ***p < 0.001 compared to the control.

**4i** analog was further investigated for its cytotoxic mechanism of action. To analyze the cell cycle changes induced by the **4i** analog, colorectal cancer cells were treated with either **4i** or DMSO as a control for 48 h and the cell were treated with PI for flow cytometric analysis. Figure 4a shows the histograms of both the control and **4i** analog demonstrating PI signals at each phase. **4i** histogram showed an increase in signal intensity at G1 phase. Moreover, the distribution of cells in each phase of the cell cycle was measured. As illustrated in Fig. 4b, **4i** analog significantly induced cell cycle arrest at G1 phase (47.62%) compared with control cells (41.93%). Hence, **4i** analog exerts its cytotoxic action against colorectal cancer via cell cycle arrest at G1 phase.

### Nortopsentin 4i analog suppressed colorectal carcinoma growth via CDK 6 inhibition

**Fig. 5:**
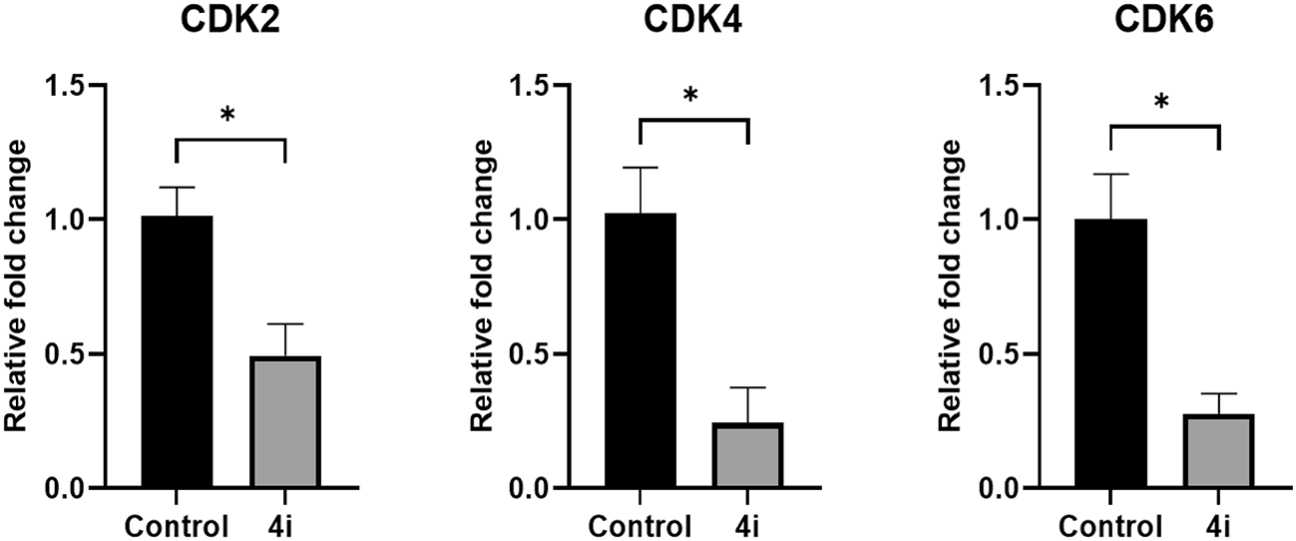
Relative gene expression of CDK2, CDK4 and CDK6 induced by **4i** analog. Data are represented as mean ± SD of three individual experiments. Statistical analysis was done using Mann Whitney test; *p < 0.05 compared to the control.

Cyclin dependent kinases are considered major key players in regulating the different phases of the cell cycle. CDK2, CDK4 and CDK6 are known to control the cell cycle transition from G1 to S phase. Since **4i** analog induced cell cycle arrest at G-1 phase, we analyzed the expression of CDK2, CDK4, and CDK6 genes. **4i** analog was able to significantly suppress the expression of all three CDK genes responsible for regulating the G1 phase as shown in Fig. 5. Such a decrease explains **4i** analog’s ability to inhibit colorectal carcinoma growth. Moreover, we measured the activity of the three CDK enzymes along with their activating cyclin subunits, CDK2/cyclin E1, CDK4/cyclin D3 and CDK6/D3. As shown in Table 1, **4i** analog significantly decreased the activity of CDK6/D3 where it’s IC_50_ (0.098 µM) compared with the positive control staurosporine which had IC_50_ of 0.277 µM. However, **4i** analog did not induce the same significant decrease in the activity of the other two enzymes. IC_50_ of CDK2/cyclin E1 was 0.658 µM compared with the positive control staurosporine with IC_50_ of 0.035 µM, while IC_50_ of CDK4/cyclin D3 was 0.265 µM compared with the positive control palbociclib with IC_50_ of 0.034 µM. Therefore, **4i** mainly inhibited colorectal carcinoma growth in CDK 6/cyclin D3 dependent manner.

**Table 1:** IC_50_ values of **4i** analog, staurosporine and palbociclib as inhibitors of different cyclin dependent kinases in colorectal carcinoma.

Type of cyclin dependent kinases	Compounds	IC50 (µM)	SD
CDK2/Cyclin E1	**4i**	0.658	± 0.037
	Staurosporine	0.035	± 0.002
CDK4/Cyclin D3	**4i**	0.265	± 0.015
	Palbociclib	0.034	± 0.002
CDK6/Cyclin D3	**4i**	0.098	± 0.004
	Staurosporine	0.277	± 0.011

### In-silico studies

#### Molecular modeling

The molecular modeling (as a theoretical method) has been done using the Gaussian09 program^29^ to analyze certain basic quantum parameters as well as the geometric shape of compound **4i**. Global interaction parameters were analyzed according to previously reported^30^ including, the highest occupied molecular orbital (E_HOMO_), the lowest unoccupied molecular orbital (E_LUMO_), energy gap (ΔE), dipole moment (µ), softness (σ), global softness (S), global electrophilicity index (ω), global hardness (η), absolute softness (S), the fraction of electrons transferred (∆N), the total energy (TE), electronegativity (χ), ionization potential (I), and electron affinity (A). The results are given in Table 2.

**Table 2:** Calculated quantum chemical parameters of compound **4i.**

Parameter	eV	Parameter	eV
E_HOMO_ (a.u.)	− 0.17307	η (a.u.)	0.035155
E_LUMO_ (a.u.)	− 0.10276	σ (a.u.) − 1	28.44546
ΔE _gap_ (a.u.)	0.07031	Pi (a.u.)	− 0.13792
µ (D)	− 9.68770	S (a.u.)^−1^	14.22273
I	0.17307	ω (a.u.)	0.27052407
A	0.10276	ΔN_max_	3.923055042
χ (a.u.)	0.137915	T.E (a.u.)	− 1314.24

**Fig. 6:**
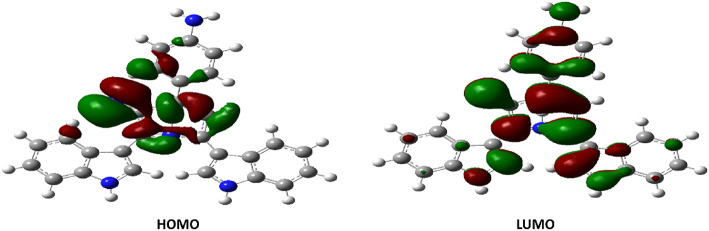
LUMO and HOMO patterns of compound **4i.**

Figure 6 displays the LUMO and HOMO molecular orbital representations of **4i**. The result from Fig. 6 showed that the HOMO was mainly distributed over the pyridine moiety and the CN functional group, with some density above the phenyl ring located at the para-position to the pyridine ring. The LUMO densities were distributed on the pyrrole moiety with its substituents (*p*-(*N,N*-dimethyl)phenyl) and CN function group. The result from Table 2 showed that the energies of HOMO and LUMO are negative, indicating that **4i** is stable. In addition, it has been proven that the value of E_HOMO_ is higher than the value of E_LUMO_ level.

**Fig. 7:**
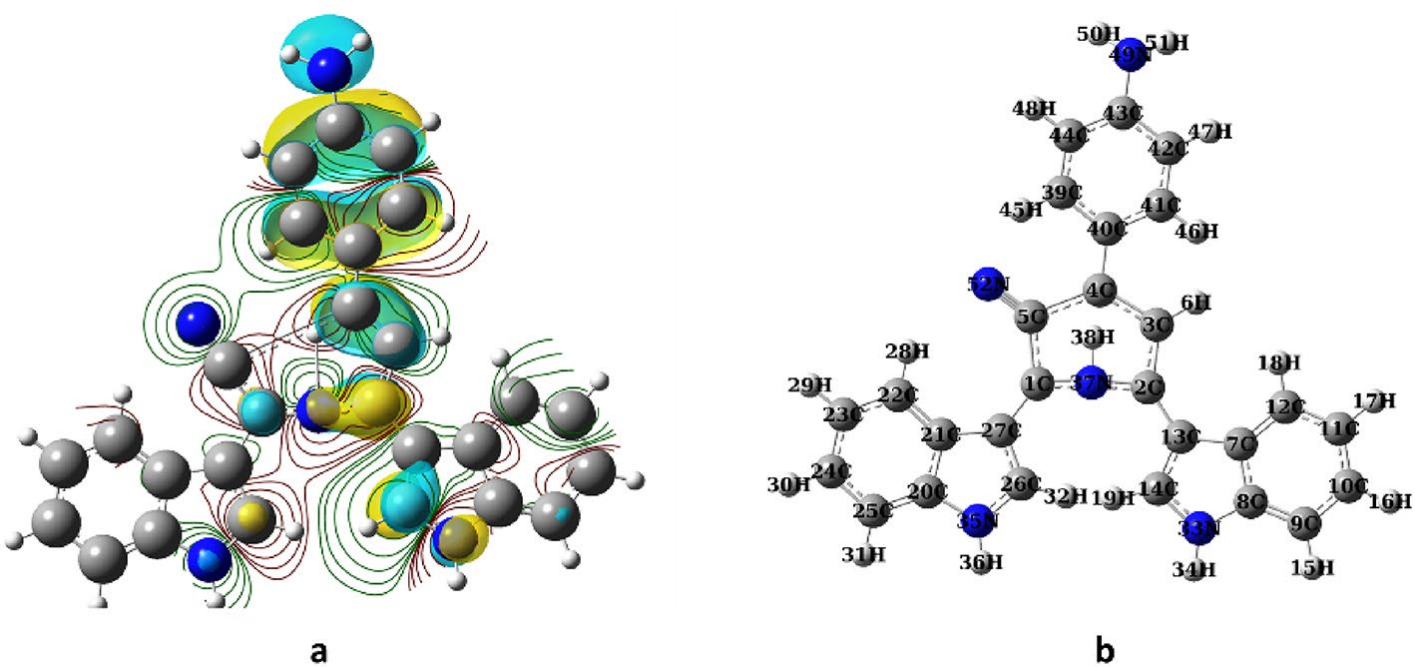
(**a**) The charge distribution of **4i**; (**b**) The numbering scheme for compound **4i** structure.

The total atomic charge distribution was estimated using charges population analysis with optimized geometry, with the results presented in Tables s2–s5 and Fig. 7a while Fig. 7b shows the numbering scheme of **4i**.

#### Drug-likeness and ADME prediction

The drug-likeness of compound **4i** according to Lipinski’s rule of five and some predictions of physicochemical and pharmacokinetic (ADME) (Table 3) using the free tool Swiss-ADME^31^, compound **4i** could have a high chance of oral bioavailability.

**Table 3:** Physicochemical and pharmacokinetic (ADME) of compound **4i**.


Physicochemical properties		Pharmacokinetics cont	
MW (g/mol)	453.54	P-gp substrate	No
HBA	2	CYP1A2 inhibitor	Yes
HBD	2	CYP2C19 inhibitor	Yes
MR	143.18	CYP2C9 inhibitor	No
TPSA (Å2)	71.50	CYP2D6 inhibitor	No
Lipophilicity		CYP3A4 inhibitor	No
Log *P*_o/w_ (MLOGP)	2.99	Drug likeness	
Water solubility		No. Lipinski violation	0 violation
Log S(ESOL)/Class	−6.74/poorly soluble	Lipinski drug-like	Yes
Log *S* (SILICOS-IT)	−11.42/insoluble	Bioavailability Score	0.55

Pharmacokinetics		Medicinal chemistry	
GI absorption	Low	PAINS	0 alert
BBB permeant	No	Brenk	0 alert
Log *K*_p_ (skin permeation)	− 4.88 cm/s	Synthetic accessibility	3.59

MW: Molecular weight ≤ 500; Log *P*_o/w_: lipophilicity (log octanol/water partition coefficient) < 4.15; HBA: Hydrogen bond acceptor ≤ 10; HBD: Hydrogen bond donor ≤ 5; MR: Molar reactivity (40 ≤ MR ≤ 130), TPSA: Topological polar surface area Å2 TPSA ≤ 140Å2, GI absorption: Gastrointestinal absorption; BBB permeant: Blood–brain barrier permeant; bioavailability score: probability of F > 10% in rat implemented; P-gp substrate: p-glycoprotein substrates; CYP: cytochrome P450; PAINS: Pan assay interference structures ; Brenk: Structural alert; Synthetic accessibility score: from 1 (very easy) to 10 (very difficult) based on 1024 fragmental contributions (FP2) modulated by size and complexity penalties trained on 12,782,590 molecules and tested on 40 external molecules (r^2^ = 0.94).

#### Molecular docking study (assessment of the binding mode of compound 4i with the target crystal structures of CDK6)

**Fig. 8:**
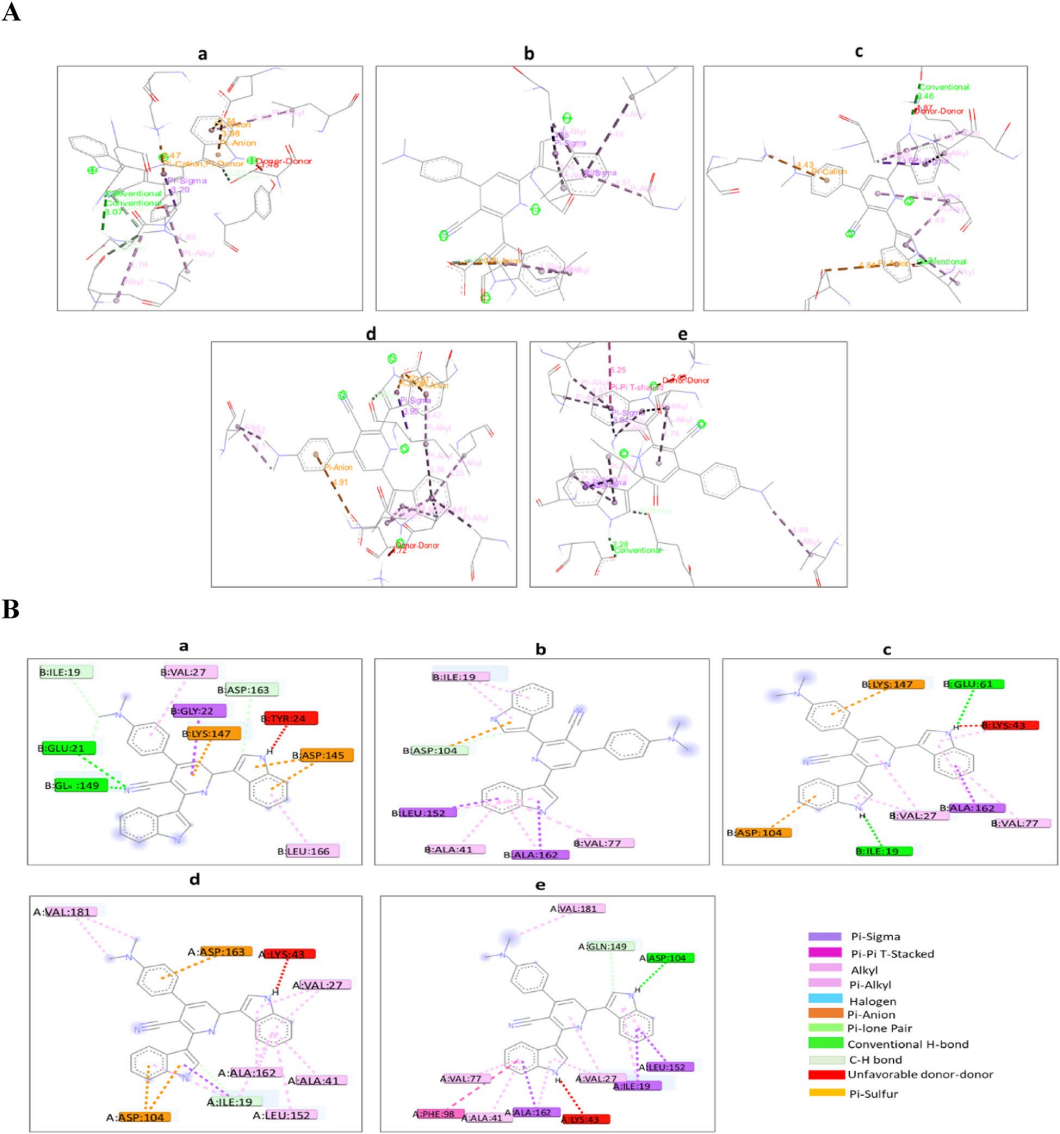
(**A**) The 2D interactions of **4i** inside the active pockets of the target crystal structures of CDK6 of PDBs: (**a**) 1XO2, (**b**) 2EUF, (**c**) 2F2C, (**d**) 3NUX, and (**e**) 4EZ5; (**B**) The 3D interactions of **4i** inside the active pockets of the target crystal structures of CDK6 of PDBs: (**a**) 1XO2, (**b**) 2EUF, (**c**) 2F2C, (**d**) 3NUX, and (**e**) 4EZ5.

The molecular docking study of compound 4i towards the target crystal structures of CDK6 binding site of PDBs: 1XO2, 2EUF, 2F2C, 3NUX, and 4EZ5 with inhibitors FSE, LQQ, AP9, 3NV, and 0RS, respectively, was achieved via PyRx tools Autodock Vina (version 1.1.2)^32^. The docking results are shown in (Figs. 8) and supplementary (Table s6, Figs. s2–s6).

Interestingly, it has been observed that the interaction of **4i** against the CDK6 crystal structures consists of some remarkable notes. Where **4i** is identical with each native ligand in many bonds with the same active amino acid of the CDK6 crystal structure. For 1XO2, compound **4i** showed conventional H-bond, C–H bond, Pi-alkyl, and Pi-sigma interactions with the amino acids, GLU21, ASP163, VAL27, GLY22 of the active pocket of 1XO2 as the native ligand (FSE) (Fig. 8a). Whereas **4i** gets engaged with the active amino acids ILE19, ALA41, and LEU125 of the active pocket of (PDB: 2EUF) via Pi-alkyl and Pi-sigma interactions as the native ligand (LQQ) (Fig. 8b). On the other hand, compound **4i** displayed Pi-alkyl and Pi-sigma interactions with the active pocket of CDK6 (2F2C) via amino acids, VAL77, ALA162, and ASP104 as native ligand (AP9) (Fig. 8c). For the active pocket of 3NUX, compound **4i** showed Pi-anion interaction with active amino acid ASP104 as the native ligand 3NV (Fig. 8d). Compound **4i** affinity towards the active pocket of 4EZ5 showed that **4i** was the most fortunate in being associated with the active amino acids ASP104, GLN49, VAL27, ILE19, LEU152, and LYS43 as the native ligand 0RS via conventional H-bond, C-H bond, Pi-alkyl, Pi-sigma, and unfavorable D-D interactions (Fig. 8e).

## Discussion

Nortopsentins are groups of sponge metabolites which are considered promising lead compounds for broad spectrum of biological properties. Nortopsentins exert cytotoxic activity against a broad range of tumors, such as breast cancer^33^, non-small cell lung cancer^21^, leukemia^20^, and liver cancer^34^. Cell cycle arrest is one of the main antiproliferative mechanisms of many antitumor agents^6^. Cyclin dependent kinases are key enzymes that orchestrate the cell cycle process and their dysregulation is a hallmark of several types of tumors^10^. To evaluate the cytotoxic activity of nortopsentin analogs (**4a**–**4j**), their antitumor activity was measured against 60 different cancer cell lines, including breast cancer, non-small cell lung cancer, prostate cancer, colorectal cancer, etc. The analog **4i** exhibited the highest level of cytotoxic activity against almost all tumor cell lines, with the highest antitumor activity against colorectal carcinoma. Therefore, we have investigated the effect of 4i analog on the cell cycle of colorectal carcinoma. Our data showed that 4i significantly induced cell cycle arrest in colorectal carcinoma at G1 phase. This is in alignment with another study that shows two nortopsentin analogs, namely thiazolyl-bis-pyrrolo[2,3-*b*]pyridines and indolyl-thiazolyl-pyrrolo[2,3-*c*]pyridines, with the ability to induce cell cycle arrest at G1 phase^22^. CDK2, CDK4, and CDK6 with their corresponding cyclins control cells transition from G1 to the S phase during the cell cycle^10,12^. Hence, the synthesis of new nortopsentin analogs which act as CDK enzymes inhibitors is a promising strategy for developing new anticancer therapeutics^35,36^. Several studies show the ability of different nortopsentin analogs to inhibit CDK1^24,25^, CDK2^37^ and CDK4^38^. Our results showed that 4i affected all three CDK enzymes that are responsible for G1 phase transition. It suppressed the expression of CDK2, CDK4 and CDK6. Moreover, it downregulated CDK6 enzymatic activity compared with the control, staurosporine. Surprisingly, there are no data about other nortopsentin analogs which target CDK6. Thus, 4i is the first nortopsentin analog to inhibit CDK6 at both transcriptional and enzymatic activity levels. Fundamental quantum parameters and geometry of compound **4i** were performed to correlate the activity of **4i** to induce the cell cycle arrest in colorectal cancer with molecular orbital (MO) energy levels of **4i**. The E_HOMO_ and E_LUMO_ molecular orbitals and their energy gap ΔE help determine the kinetic stability and chemical reactivity of the molecule when it interacts with the target enzyme or protein^29^. The result showed that the energies of HOMO and LUMO were negative, which indicates **4i** is stable. An increase in the value of the E_HOMO_ level and a decrease in the value of the E_LUMO_ level show an increment in the binding ability between **4i** and the target enzyme active pocket. As ΔE decreases, the reactivity of the molecule increases, leading to a better inhibition efficiency^39^. A molecule with a low energy gap is more polarizable and is generally associated to the high chemical activity and low kinetic stability, such molecule is called “soft molecule”^39^. Our results indicated that **4i** is a soft molecule that has a small ΔE gap (0.07031 eV). High values of the ionization energy (I) evidence the chemical inertness and strong stability, whereas small ionization energy denotes the high reactivity of the atoms and molecules^40^. Our results showed that compound **4i** had low ionization energy (0.17307 eV) indicating its activity. **4i** also showed high chances of oral bioavailability due to its compatibility with Lipinski’s rule of five. Moreover, to correlate the in vitro CDK6 data for the present analog (**4i**), the target crystal structures of CDK6 binding site of PDBs: 1XO2, 2EUF, 2F2C, 3NUX, and 4EZ5 with the inhibitors FSE, LQQ, AP9, 3NV, and 0RS, respectively was employed using the molecular docking study. Our results indicated that **4i** was superimposable to the native ligands. In addition, it showed good binding mode with the active pocket through the formation of multiple interactions with the key amino acids of CDK6 in a similar manner as native ligands. Also, it revealed a good binding energies towards the binding site for all CDK6 crystal structures with values higher than the native ligands. Hence, **4i** analog is the first nortopsentin analog that acts as antitumor agent against colorectal carcinoma via inhibiting CDK6 at both transcriptional and enzymatic activity levels. Despite the struggle to find explicit structure–activity relationships from the biological data, some conclusions can still be considered. First, nortopsintine with no substitution (R = H) or substituted with a, halogen (R = Br, Cl, F); b, (R = OMe, di,-OMe, tri-OMe); c, (R = NO_2_); d, (R = OH) doesn’t show antiproliferative activity. Second, only nortopsintine substituted with N(CH_3_)_2_ (nitrogen groups with lone pairs representing an activating group) enhanced antiproliferative activity against most cancer cell lines under study. This finding is consistent with previous studies that reported that the presence of an electron-donating group on the phenyl ring is largely preferred over the presence of an electron-withdrawing group, negatively affecting cytotoxic efficacy^41^.

## Materials and methods

### Cell culture

HCT-116 (colon cancer cell line) was obtained from the American Type Culture Collection (ATCC). The cancer cell line was grown in Dulbecco’s Modified Eagle’s Medium (DMEM) supplemented with 10% fetal bovine serum (FBS), penicillin/streptomycin (100 U/mL and 100 μg/mL, respectively) and 2 mM l-glutamine. The cells were maintained at 37 °C in 5% CO_2_.

### Cell proliferation and IC_50_ value determination

The cytotoxic activity of nortopsintin analogs (**4a–4j**) was determined by the national cancer institute (NCI, Bethesda, Maryland, USA) against 60 different cancer cell lines at a single concentration of (10^−5^ M). For the determination of IC_50_ value, we used MTT assay kit (Sigma) according to the manufacturer’s protocol. HCT-116 cells were seeded into a 96-well plate at a concentration of 20,000 cells/well. After 24 h, the media was aspirated and replaced with serum-free media containing serial dilutions of the **4i** compound. The cells were treated for 48 h in triplicates. 0.5% dimethyl sulfoxide (DMSO) was used as negative control and staurosporine was used as positive control. The percentage of cytotoxicity was calculated using the following equation: % Cytotoxicity = [1 − (AVx/AVNC)] × 100.

Where, AVx denotes the average absorbance of the sample and AVNC denotes the average absorbance of the negative control. The absorbance was measured at 570 nm with reference at 690 nm.

### RNA isolation

The cells were seeded in a six-well plate at a concentration of 3 × 10^5^ cells/well. After a 24 h incubation, the IC_50_ dose of active compound **4i** was applied to each well for 48 h. The cells were collected and centrifuged at 2500 rpm for 15 min at 4 °C. The total RNA isolation kit (RNeasy extraction kit—QIAGEN) was used to isolate RNA according to the manufacturer’s instructions.

Gene expression analysis by quantitative real-time PCR. The levels of gene expression of CDK2, CDK4 and CDK6 were determined using iScript One-Step RT-PCR kit with SYBR Green (BIORAD) according to the manufacturer protocol. The sequences of the used primers are illustrated in Table 4. The genes expression was normalized to GAPDH as the housekeeping gene. The protocol used for cDNA synthesis was: 10 min at 50 °C, iScript Reverse transcriptase inactivation: 5 min at 95 °C and PCR cycling and detection (30 to 45 cycles): 10 s at 95 °C and 30 s at 55 °C to 60 °C.

**Table 4:** Primers sequence of different genes.

Gene	Primer sequence
CDK2	F: 5′-ATGGATGCCTCTGCTCTCACTG-3′ R: 5′-CCCGATGAGAATGGCAGAAAGC-3′
CDK4	F: 5′-CCATCAGCACAGTTCGTGAGGT-3′ R: 5′-TCAGTTCGGGATGTGGCACAGA-3′
CDK6	F: 5′-GGATAAAGTTCCAGAGCCTGGAG-3′ R: 5′-GCGATGCACTACTCGGTGTGAA-3′
GAPDH	F: 5′-GTCTCCTCTGACTTCAACAGCG-3′ R: 5′-ACCACCCTGTTGCTGTAGCCAA-3′

### Cell cycle analysis by FACS analysis

Briefly, HCT116 cells were treated with **4i** compound’s IC_50_ for 48 h. Then, the cells were fixed in cold 70% ethanol solution for 2 h at 4 °C and centrifuged at 800 g for 5 min. The ethanol solution was removed. PBS was used to wash the cells twice. DNA was stained with 1 µg/mL propidium iodide (PI) solution (Sigma-Aldrich, St. Louis, MO, USA), supplemented with 100 µg/mL RNase A (Roche, Basel, Switzerland) for 30 min in the dark at 37 °C. After centrifugation, the cells were suspended in phosphate buffer solution and the fluorescence was measured using flow cytometry by FACSCalibur (BD Biosciences, Mountain View, CA). The data were analyzed using the CellQuest software (BD Biosciences).

### Cyclin dependent kinases enzymatic activity assay

HCT-116 cells were treated with **4i** or the control inhibitors: palbociclib and staurosporine for 48 h in triplicates. ADP-Glo™ Kinase Assay kit (Promega, USA) was used for the enzymatic activity analysis according to the manufacturer’s instructions. In brief, to each well we added 1 μl of inhibitor, 2 μl of each cyclin dependent kinase enzymes, and 2 μl of substrate/ATP mix and incubated at room temperature for 60 min. After that 5 μl of ADP‐Glo™ reagent was added and incubated at room temperature for 40 min. 10 μl of kinase detection reagent was added and incubated at room temperature for 30 min, then the luminescence was measured.

### In silico study

#### Computational methodology

With Gaussian09 software, the ideal structural geometry of compound **4i** was calculated using the ground state, DFT, B3LYP, and 3-211G. Gaussian files were shown using the molecular visualization software Gauss View^42^. The DFT/B3LYP quantum chemical parameters were computed using HOMO–LUMO energies that agreed with the numerical pattern shown in the gas phase compounds view. In optimized structures, significant bond lengths, bond angles, dihedral angles, charges, and excitation energy for coordinating groups were also calculated.

#### Molecular docking

Molecular docking studies of the active compound **4i** against the crystal structures of five CDK6 targets were performed using PyRx tools Autodock Vina (version 1.1.2)^32^. The crystal structures of CDK6 complexed with the inhibitors were downloaded from RCSB Protein Data Bank (accessed on 29-6-2023). The molecular interactions and binding modes of the top poses were visually examined using BIOVIA Discovery Studio 2021.

#### Statistics

Comparisons between multiple treatments were made by either, Student *t*-test, one-way or two-way ANOVA, followed by Dunnett’s multiple comparison test; *p < 0.05, **p < 0.01, ***p < 0.001 were considered to be a significant difference. GraphPad Prism 8 (GraphPad Software, San Diego, CA, USA) was used for statistical analysis and plotting the graphs.

## Conclusions

Nortopsentin analogs **4a–j** were screened against 60 different cancer cell lines. **4i** analog exhibited the highest level of cytotoxic activity against almost all tumor cell lines including colorectal carcinoma. **4i** induced cell cycle arrest in colorectal carcinoma through downregulating the expression of CDK2, CDK4 and CDK6. In addition, **4i** decreased the enzymatic activity of CDK6 compared with the control staurosporine. The theoretical study of some basic quantum factors and the geometric shape of compound **4i** showed that it’s a stable and soft molecule. It showed negative E_HOMO_ and E_LUMO_ energies and a small ∆E gap. **4i** has also revealed high potential for oral bioavailability due to its compatibility with Lipinski’s rule of five. Furthermore, **4i** showed good binding mode with CDK6 active pockets through the formation of multiple interactions with the key amino acids in a similar manner as native ligands. Hence, **4i** analog is a promising candidate for further preclinical studies against colorectal carcinoma.

## Electronic supplementary material

Below is the link to the electronic supplementary material.

Supplementary Material 1

## Data Availability

All data generated or analyzed during this study are included in this published article and its supplementary information file.
