# Supplementary material for: Anti colorectal cancer activity and in silico studies of novel pyridine nortopsentin analog as cyclin dependent kinase 6 inhibitor
Source: Sci Rep. 2024 Nov 1;14:26327. doi: 10.1038/s41598-024-75411-3 (PMC11530689; doi:10.1038/s41598-024-75411-3)
Supplement: Supplementary file 1 — Supplementary Material 1 [file 41598_2024_75411_MOESM1_ESM.docx]

| **Table S1.** Growth percentage of nortopsintin analogs against 60 cancer cell lines. | | | | | | | | | | |
| --- | --- | --- | --- | --- | --- | --- | --- | --- | --- | --- |
| **Panel/**  **Cell line** | **Growth %** | | | | | | | | | |
|  | **4a** | **4b** | **4c** | **4d** | **4e** | **4f** | **4g** | **4h** | **4i** | **4j** |
| **Leukemia** | | | | | | | | | | |
| CCRF-CEM | 102.65 | 102.80 | 101.97 | 102.09 | 101.65 | 97.196 | 99.17 | 103.06 | 78.55 | 76.51 |
| HL-60 (TB) | 103.72 | 102.74 | 102.90 | 98.89 | 106.85 | 101.74 | 101.32 | 97.00 | 85.35 | 107.76 |
| K-562 | 100.60 | 96.82 | 97.57 | 104.54 | 96.99 | 93.22 | 90.08 | 94.26 | 39.74 | 67.98 |
| MOLT-4 | 102.45 | 99.47 | 99.33 | 103.02 | 94.10 | 98.62 | 100.72 | 100.27 | 98.39 | 87.60 |
| RPMI-8226 | 100.70 | 102.03 | 102.27 | 100.93 | 104.41 | 98.96 | 97.18 | 95.92 | 69.39 | 101.54 |
| SR | 96.68 | 94.65 | 98.46 | 89.11 | 89.59 | 81.27 | 94.92 | 84.51 | 32.38 | 76.19 |
| **Non-Small Cell Lung Cancer** | | | | | | | | | | |
| A549/ATCC | 98.73 | 98.58 | 100.29 | 101.42 | 99.97 | 98.51 | 99.60 | 96.38 | 89.56 | 105.12 |
| EKVX | 83.07 | 85.89 | 87.50 | 92.76 | 91.11 | 91.45 | 87.27 | 82.20 | 93.75 | 100.08 |
| HOP-62 | 92.66 | 98.17 | 93.62 | 100.69 | 90.00 | 96.31 | 87.93 | 99.88 | 82.30 | 94.38 |
| HOP-92 | 92.10 | 92.57 | 99.01 | 93.44 | 82.07 | 95.02 | 92.84 | 98.33 | 104.62 | 125.42 |
| NCI-H226 | 89.89 | 87.72 | 96.15 | 94.04 | 92.14 | 87.28 | 87.22 | 87.03 | 79.62 | 86.10 |
| NCI-H23 | 95.64 | 93.93 | 89.48 | 100.43 | 103.56 | 103.68 | 93.03 | 97.26 | 88.40 | 89.30 |
| NCI-H322M | 105.41 | 104.62 | 103.02 | 106.25 | 108.08 | 104.89 | 93.11 | 95.29 | 92.92 | 101.04 |
| NCI-H460 | 106.27 | 106.47 | 106.15 | 104.14 | 107.18 | 103.37 | 106.65 | 100.56 | 60.31 | 103.74 |
| NCI-H522 | 97.10 | 94.73 | 96.57 | 97.04 | 92.71 | 92.06 | 93.33 | 95.41 | 93.15 | 85.59 |
| **Colon Cancer** | | | | | | | | | | |
| COLO205 | 108.26 | 111.43 | 106.5 | 113.48 | 102.89 | 110.17 | 106.57 | 114.22 | 91.18 | 111.37 |
| HCC-2998 | 93.57 | 114.86 | 117.80 | 109.40 | 128.75 | 112.62 | 107.87 | 108.98 | 88.23 | 126.05 |
| HCT-116 | 103.11 | 104.37 | 106.26 | 104.67 | 106.07 | 100.81 | 104.41 | 100.64 | 57.78 | 105.38 |
| HCT-15 | 99.26 | 97.86 | 98.27 | 99.59 | 89.77 | 101.68 | 100.84 | 99.04 | 44.65 | 57.19 |
| HT29 | 102.69 | 107.82 | 107.41 | 110.19 | 105.49 | 108.12 | 105.35 | 111.20 | 63.02 | 111.48 |
| KM12 | 102.51 | 99.86 | 102.73 | 100.66 | 96.65 | 98.72 | 99.69 | 98.09 | 58.37 | 96.89 |
| SW-620 | 101.78 | 105.18 | 106.55 | 106.63 | 113.43 | 102.51 | 100.40 | 105.72 | 46.63 | 108.39 |
| **CNS Cancer** | | | | | | | | | | |
| SF-268 | 96.15 | 100.28 | 103.64 | 101.12 | 105.10 | 99.97 | 97.03 | 93.92 | 96.47 | 105.02 |
| SF-295 | 96.36 | 99.37 | 99.60 | 102.68 | 95.83 | 101.07 | 95.05 | 100.99 | 79.83 | 87.00 |
| SF-539 | 98.60 | 97.32 | 98.85 | 100.06 | 93.61 | 94.25 | 93.66 | 97.59 | 75.80 | 66.62 |
| SNB-19 | 97.62 | 98.35 | 101.34 | 102.08 | 102.42 | 96.57 | 98.31 | 100.62 | 79.65 | 108.35 |
| SNB-75 | 81.21 | 91.48 | 100.87 | 98.55 | 97.63 | 87.63 | 77.85 | 90.31 | 86.73 | 90.85 |
| U251 | 94.26 | 94.64 | 99.27 | 94.85 | 102.14 | 99.96 | 100.17 | 99.87 | 92.50 | 96.90 |
| **Melanoma** | | | | | | | | | | |
| LOX IMVI | 100.06 | 99.48 | 101.39 | 107.36 | 86.06 | 82.70 | 98.85 | 92.89 | 70.02 | 62.75 |
| MALME-3M | 99.77 | 103.25 | 107.80 | 101.85 | 113.82 | 108.42 | 88.70 | 102.24 | 97.41 | 114.20 |
| M14 | 100.81 | 102.89 | 102.44 | 97.40 | 93.75 | 94.97 | 98.40 | 97.98 | 97.66 | 100.26 |
| MDA-MB-435 | 102.63 | 108.30 | 106.56 | 107.27 | 104.98 | 99.41 | 100.31 | 104.27 | 81.62 | 90.35 |
| SK-MEL-2 | 97.65 | 95.21 | 103.64 | 96.13 | 100.09 | 88.37 | 92.02 | 96.75 | 102.08 | 100.11 |
| SK-MEL-28 | 109.51 | 112.22 | 103.30 | 106.51 | 108.94 | 100.94 | 107.46 | 106.75 | 101.89 | 76.45 |
| SK-MEL-5 | 99.18 | 97.46 | 99.39 | 100.33 | 97.12 | 98.48 | 98.25 | 97.67 | 96.50 | 84.85 |
| UACC-257 | 106.98 | 107.82 | 108.88 | 107.07 | 107.78 | 112.07 | 104.77 | 104.66 | 101.12 | 108.53 |
| UACC-62 | 94.92 | 94.51 | 89.22 | 98.33 | 95.53 | 93.55 | 87.61 | 85.82 | 85.78 | 96.73 |
| **Ovarian Cancer** | | | | | | | | | | |
| IGROV1 | 82.09 | 73.65 | 91.10 | 102.10 | 85.18 | 93.71 | 74.20 | 81.21 | 74.28 | 76.43 |
| OVCAR-3 | 109.77 | 102.43 | 107.09 | 102.48 | 96.29 | 99.19 | 102.73 | 97.76 | 91.03 | 92.93 |
| OVCAR-4 | 103.65 | 102.16 | 106.75 | 101.04 | 106.10 | 101.53 | 103.83 | 99.28 | 95.26 | 94.07 |
| OVCAR-5 | 103.05 | 107.40 | 105.15 | 109.33 | 103.69 | 108.89 | 103.55 | 105.31 | 102.79 | 108.44 |
| OVCAR-8 | 98.73 | 96.50 | 101.97 | 100.20 | 98.49 | 102.00 | 98.26 | 98.23 | 96.39 | 99.60 |
| NCI/ADR-RES | 98.30 | 99.61 | 98.53 | 103.38 | 108.38 | 97.14 | 100.17 | 96.36 | 90.91 | 102.41 |
| SK-OV-3 | 93.31 | 100.81 | 91.24 | 96.90 | 94.59 | 99.89 | 97.19 | 90.72 | 94.88 | 96.54 |
| **Renal Cancer** | | | | | | | | | | |
| 786-0 | 102.19 | 101.55 | 102.38 | 99.50 | 102.90 | 104.74 | 102.99 | 106.06 | 99.08 | 101.37 |
| A498 | 98.67 | 95.35 | 97.38 | 89.44 | 108.69 | 96.67 | 103.11 | 99.31 | 98.45 | 110.66 |
| ACHN | 96.37 | 99.45 | 101.81 | 101.89 | 95.12 | 97.24 | 98.52 | 94.69 | 98.60 | 72.15 |
| CAKI-1 | 87.26 | 81.43 | 87.84 | 92.51 | 84.47 | 91.03 | 75.97 | 82.68 | 68.37 | 90.60 |
| RXF 393 | 94.99 | 109.74 | 114.27 | 111.59 | 94.44 | 107.63 | 120.31 | 111.16 | 106.90 | 83.24 |
| SN12C | 95.61 | 96.36 | 97.84 | 98.76 | 99.19 | 95.11 | 91.09 | 98.48 | 92.07 | 95.63 |
| TK-10 | 123.79 | 146.78 | 138.81 | 127.59 | 141.73 | 124.45 | 128.09 | 142.08 | 107.28 | 149.07 |
| UO-31 | 68.28 | 71.05 | 70.21 | 84.72 | 74.78 | 71.31 | 65.92 | 70.38 | 72.88 | 78.37 |
| **Prostate Cancer** | | | | | | | | | | |
| PC-3 | 92.44 | 93.93 | 100.13 | 103.05 | 89.91 | 93.60 | 91.47 | 93.76 | 89.56 | 98.38 |
| DU-145 | 108.34 | 107.37 | 108.08 | 106.26 | 108.02 | 101.42 | 106.13 | 103.04 | 99.65 | 104.31 |
| **Breast Cancer** | | | | | | | | | | |
| MCF7 | 94.97 | 85.19 | 89.99 | 89.33 | 90.48 | 90.74 | 88.35 | 90.39 | 49.30 | 87.55 |
| MDA-MB-231/ATCC | 92.64 | 101.07 | 97.80 | 102.47 | 102.65 | 100.79 | 82.84 | 96.84 | 99.81 | 92.33 |
| HS 578T | 93.45 | 97.14 | 100.65 | 95.18 | 100.69 | 102.35 | 99.50 | 94.79 | 104.13 | 89.18 |
| BT-549 | 94.69 | 103.55 | 113.43 | 108.84 | 97.48 | 103.60 | 101.50 | 108.68 | 103.60 | 96.81 |
| T-47D | 86.69 | 85.37 | 100.54 | 89.92 | 86.61 | 98.31 | 96.11 | 88.78 | 95.86 | 98.31 |
| MDA-MB-468 | 93.05 | 104.30 | 102.95 | 99.29 | 102.09 | 92.23 | 93.79 | 92.34 | 81.79 | 101.94 |

| **Table s2.** Selected geometric atomic charges | | | | | |
| --- | --- | --- | --- | --- | --- |
| **Atom No.** | **Atomic charges** | **Atom No.** | **Atomic charges** | **Atom No.** | **Atomic charges** |
| C1 | 0.533119 | H6 | 0.169649 | H47 | 0.147281 |
| C2 | 0.446906 | H28 | 0.219335 | H48 | 0.151732 |
| C3 | -0.573636 | H15 | 0.163542 | N49 | -0.782346 |
| C4 | 0.267753 | H16 | 0.153524 | H50 | 0.287435 |
| C5 | -0.647293 | H17 | 0.15133 | H51 | 0.283731 |
| C7 | -0.190907 | H18 | 0.193686 | H38 | -0.028684 |
| C8 | 0.265242 | H19 | 0.121256 | C39 | -0.045809 |
| C9 | -0.086642 | H29 | 0.150301 | C40 | -0.026278 |
| C10 | -0.11847 | H30 | 0.146256 | C41 | -0.073408 |
| C11 | -0.211158 | H31 | 0.156086 | C42 | -0.195309 |
| C12 | 0.085548 | H32 | 0.117384 | H47 | 0.147281 |
| C13 | -0.039364 | N33 | -0.835969 | H48 | 0.151732 |
| C14 | 0.324105 | H34 | 0.333419 | N49 | -0.782346 |
| C24 | -0.11965 | N35 | -0.834665 | H50 | 0.287435 |
| C25 | -0.095155 | H36 | 0.331414 | H51 | 0.283731 |
| C26 | 0.32063 | N37 | -0.73062 | N52 | -0.206272 |
| C27 | -0.039732 |  |  |  |  |

| **Table s3.** Selected geometric bond length | | | | | |
| --- | --- | --- | --- | --- | --- |
|  | **Bond length** |  | **Bond length** |  | **Bond length** |
| R(1,5) | 1.3756 | R(10,16) | 1.0699 | R(33,34) | 1 |
| R(1,27) | 1.5395 | R(11,12) | 1.4092 | R(35,36) | 1 |
| R(1,37) | 1.1807 | R(11,17) | 1.0701 | R(37,38) | 0.9999 |
| R(2,3) | 1.3754 | R(12,18) | 1.0701 | R(39,40) | 1.4013 |
| R(2,13) | 1.5395 | R(13,14) | 1.4098 | R(39,44) | 1.4013 |
| R(2,37) | 1.1807 | R(14,19) | 1.07 | R(39,45) | 1.0701 |
| R(3,4) | 1.5438 | R(14,33) | 1.3585 | R(40,41) | 1.4012 |
| R(3,6) | 1.0701 | R(19,32) | 0.92 | R(41,42) | 1.4009 |
| R(3,38) | 1.3585 | R(20,21) | 1.3666 | R(41,46) | 1.07 |
| R(4,5) | 1.5441 | R(20,25) | 1.3464 | R(42,43) | 1.4012 |
| R(4,37) | 1.7902 | R(20,35) | 1.4752 | R(42,47) | 1.07 |
| R(4,38) | 0.7903 | R(21,22) | 1.3454 | R(43,44) | 1.4015 |
| R(4,40) | 1.5394 | R(21,27) | 1.5241 | R(43,49) | 1.4698 |
| R(5,38) | 1.3588 | R(22,23) | 1.4094 | R(44,48) | 1.0699 |
| R(5,52) | 1.1466 | R(22,28) | 1.07 | R(45,52) | 1.9544 |
| R(7,8) | 1.3664 | R(23,24) | 1.4187 | R(49,50) | 1 |
| R(7,12) | 1.3453 | R(23,29) | 1.0699 | R(49,51) | 1.0001 |
| R(7,13) | 1.5237 | R(24,25) | 1.4105 | R(26,35) | 1.3583 |
| R(8,9) | 1.3464 | R(24,30) | 1.0699 | R(10,11) | 1.4185 |
| R(8,33) | 1.4754 | R(25,31) | 1.07 | R(26,32) | 1.07 |
| R(9,10) | 1.4106 | R(26,27) | 1.4096 | R(9,15) | 1.07 |

| **Table s4.** Selected geometric bond angles (°) | | | | | |
| --- | --- | --- | --- | --- | --- |
|  | **Bond Angles (°)** |  | **Bond Angles (°)** |  | **Bond Angles (°)** |
| A(5,1,27) | 137.9486 | A(12,7,13) | 130.368 | A(21,20,25) | 122.3415 |
| A(5,1,37) | 84.1608 | A(7,8,9) | 122.3184 | A(21,20,35) | 108.2287 |
| A(27,1,37) | 137.8905 | A(7,8,33) | 108.2226 | A(25,20,35) | 129.4298 |
| A(3,2,13) | 137.8758 | A(9,8,33) | 129.459 | A(20,21,22) | 123.1128 |
| A(3,2,37) | 84.1507 | A(8,9,10) | 117.5549 | A(20,21,27) | 106.4778 |
| A(13,2,37) | 137.9735 | A(8,9,15) | 121.2186 | A(22,21,27) | 130.4094 |
| A(2,3,4) | 109.4034 | A(10,9,15) | 121.2266 | A(21,22,23) | 117.3098 |
| A(2,3,6) | 125.2968 | A(9,10,11) | 119.9048 | A(21,22,28) | 121.3448 |
| A(2,3,38) | 78.6414 | A(9,10,16) | 120.0471 | A(23,22,28) | 121.3454 |
| A(4,3,6) | 125.2998 | A(11,10,16) | 120.0482 | A(22,23,24) | 119.7829 |
| A(6,3,38) | 156.0615 | A(10,11,12) | 119.7729 | A(22,23,29) | 120.111 |
| A(3,4,5) | 123.1031 | A(10,11,17) | 120.1183 | A(24,23,29) | 120.1061 |
| A(3,4,37) | 61.5477 | A(12,11,17) | 120.1087 | A(23,24,25) | 119.9023 |
| A(3,4,40) | 118.3756 | A(7,12,11) | 117.3153 | A(23,24,30) | 120.0566 |
| A(5,4,37) | 61.5555 | A(7,12,18) | 121.3393 | A(25,24,30) | 120.0411 |
| A(5,4,40) | 118.5213 | A(11,12,18) | 121.3455 | A(20,25,24) | 117.5507 |
| A(1,5,4) | 109.3801 | A(2,13,7) | 126.7033 | A(20,25,31) | 121.2195 |
| A(1,5,38) | 78.624 | A(2,13,14) | 126.8191 | A(24,25,31) | 121.2298 |
| A(1,5,52) | 125.2953 | A(7,13,14) | 106.4776 | A(27,26,32) | 125.2187 |
| A(4,5,52) | 125.3246 | A(13,14,19) | 125.2397 | A(27,26,35) | 109.544 |
| A(38,5,52) | 156.0803 | A(13,14,33) | 109.5269 | A(32,26,35) | 125.2372 |
| A(8,7,12) | 123.1337 | A(19,14,33) | 125.2334 | A(1,27,21) | 126.8131 |
| A(8,7,13) | 106.4983 | A(14,19,32) | 164.604 | A(1,27,26) | 126.716 |
| A(21,27,26) | 106.4708 | A(4,40,41) | 119.9207 | A(44,39,45) | 120.0165 |
| A(19,32,26) | 165.3385 | A(39,40,41) | 120.0319 | A(4,40,39) | 120.0474 |
| A(8,33,14) | 109.2746 | A(40,41,42) | 120.0004 | A(4,40,41) | 119.9207 |
| A(8,33,34) | 125.3698 | A(40,41,46) | 119.9956 | A(43,49,50) | 109.4628 |
| A(14,33,34) | 125.3556 | A(42,41,46) | 120.004 | A(43,49,51) | 109.4547 |
| A(20,35,26) | 109.2787 | A(41,42,43) | 119.9894 | A(50,49,51) | 109.4861 |
| A(20,35,36) | 125.3693 | A(41,42,47) | 119.9984 | A(5,38,37) | 92.3129 |
| A(26,35,36) | 125.352 | A(43,42,47) | 120.0122 | A(40,39,44) | 119.967 |
| A(1,37,2) | 150.1981 | A(42,43,44) | 120.0189 | A(40,39,45) | 120.0165 |
| A(1,37,4) | 104.9036 | A(42,43,49) | 119.9707 | A(39,44,48) | 120.0094 |
| A(1,37,38) | 104.902 | A(44,43,49) | 120.0103 | A(43,44,48) | 119.9983 |
| A(2,37,4) | 104.8982 | A(39,44,43) | 119.9924 | A(3,38,37) | 92.3076 |
| A(2,37,38) | 104.8999 |  |  |  |  |

| **Table s5.** Selected geometric Dihedral Bond angles (°) | | | | | |
| --- | --- | --- | --- | --- | --- |
|  | **Dihedral Bond angles (°)** |  | **Dihedral Bond angles (°)** |  | **Dihedral Bond angles (°)** |
| D(1,5,38,37) | -0.1247 | D(9,8,33,14) | -179.9943 | D(13,14,33,8) | -0.007 |
| D(52,5,38,37) | -179.8423 | D(9,8,33,34) | -0.0003 | D(13,14,33,34) | 179.9989 |
| D(12,7,8,9) | -0.0123 | D(8,9,10,11) | 0.0051 | D(19,14,33,8) | 179.9976 |
| D(12,7,8,33) | 179.9819 | D(8,9,10,16) | -179.9936 | D(19,14,33,34) | 0.0035 |
| D(13,7,8,9) | 179.9943 | D(15,9,10,11) | -179.9893 | D(14,19,32,26) | -0.1324 |
| D(13,7,8,33) | -0.0114 | D(15,9,10,16) | 0.012 | D(25,20,21,22) | 0.0149 |
| D(8,7,12,11) | 0.0136 | D(9,10,11,12) | -0.0034 | D(25,20,21,27) | -179.9909 |
| D(8,7,12,18) | -179.9843 | D(9,10,11,17) | 179.9916 | D(35,20,21,22) | -179.9783 |
| D(13,7,12,11) | -179.9948 | D(16,10,11,12) | 179.9953 | D(35,20,21,27) | 0.016 |
| D(13,7,12,18) | 0.0073 | D(16,10,11,17) | -0.0097 | D(21,20,25,24) | -0.0161 |
| D(8,7,13,2) | -179.991 | D(10,11,12,7) | -0.0057 | D(21,20,25,31) | 179.9876 |
| D(8,7,13,14) | 0.0075 | D(10,11,12,18) | 179.9922 | D(35,20,25,24) | 179.9755 |
| D(12,7,13,2) | 0.0163 | D(17,11,12,7) | 179.9993 | D(35,20,25,31) | -0.0208 |
| D(12,7,13,14) | -179.9852 | D(17,11,12,18) | -0.0028 | D(21,20,35,26) | -0.0001 |
| D(7,8,9,10) | 0.0024 | D(2,13,14,19) | -0.0061 | D(21,20,35,36) | -179.9919 |
| D(7,8,9,15) | 179.9967 | D(2,13,14,33) | 179.9985 | D(25,20,35,26) | -179.9926 |
| D(33,8,9,10) | -179.9905 | D(7,13,14,19) | 179.9954 | D(25,20,35,36) | 0.0156 |
| D(33,8,9,15) | 0.0039 | D(7,13,14,33) | 0 | D(20,21,22,23) | -0.0042 |
| D(7,8,33,14) | 0.012 | D(13,14,19,32) | -0.507 | D(20,21,22,28) | 179.9945 |
| D(7,8,33,34) | -179.9939 | D(33,14,19,32) | 179.4877 | D(27,21,22,23) | -179.997 |
| D(1,5,38,37) | -0.1247 | D(9,8,33,14) | -179.9943 | D(27,21,22,28) | 0.0017 |
| D(52,5,38,37) | -179.8423 | D(9,8,33,34) | -0.0003 | D(20,21,27,1) | 179.9751 |
| D(12,7,8,9) | -0.0123 | D(8,9,10,11) | 0.0051 | D(13,14,33,8) | -0.007 |
| D(7,1,5,4) | 179.9657 | D(37,2,3,4) | 0.0149 | D(6,3,38,37) | 179.8673 |
| D(27,1,5,38) | -179.9033 | D(37,2,3,6) | -179.9807 | D(3,4,5,1) | -0.0576 |
| D(27,1,5,52) | -0.0436 | D(37,2,3,38) | -0.1122 | D(3,4,5,52) | 179.9517 |
| D(37,1,5,4) | -0.0249 | D(3,2,13,7) | 0.1072 | D(37,4,5,1) | 0.0186 |
| D(37,1,5,38) | 0.1061 | D(3,2,13,14) | -179.891 | D(37,4,5,52) | -179.9721 |
| D(37,1,5,52) | 179.9658 | D(37,2,13,7) | -179.8715 | D(40,4,5,1) | 179.9333 |
| D(5,1,27,21) | -0.139 | D(37,2,13,14) | 0.1303 | D(40,4,5,52) | -0.0574 |
| D(5,1,27,26) | 179.8627 | D(3,2,37,1) | -179.8056 | D(3,4,37,1) | 179.9062 |
| D(37,1,27,21) | 179.8471 | D(3,2,37,4) | -0.0126 | D(3,4,37,2) | 0.0127 |
| D(37,1,27,26) | -0.1512 | D(3,2,37,38) | 0.1546 | D(5,4,37,1) | -0.0212 |
| D(5,1,37,2) | 179.814 | D(13,2,37,1) | 0.1801 | D(5,4,37,2) | -179.9147 |
| D(5,1,37,4) | 0.021 | D(13,2,37,4) | 179.9731 | D(1,37,40,39) | 0.0081 |
| D(5,1,37,38) | -0.1462 | D(13,2,37,38) | -179.8597 | D(1,37,40,41) | -179.9316 |
| D(27,1,37,2) | -0.1767 | D(2,3,4,5) | 0.0651 | D(2,37,40,39) | -179.9038 |
| D(27,1,37,4) | -179.9696 | D(2,3,4,37) | -0.0111 | D(2,37,40,41) | 0.1565 |
| D(27,1,37,38) | 179.8632 | D(2,3,4,40) | -179.9259 | D(3,4,40,39) | -179.9246 |
| D(13,2,3,4) | -179.9707 | D(6,3,4,5) | -179.9393 | D(3,4,40,41) | 0.0893 |
| D(13,2,3,6) | 0.0337 | D(6,3,4,37) | 179.9844 | D(5,4,40,39) | 0.084 |
| D(13,2,3,38) | 179.9021 | D(6,3,4,40) | 0.0697 | D(5,4,40,41) | -179.9021 |
| D(37,2,3,4) | 0.0149 | D(2,3,38,37) | 0.1319 | D(1,5,38,37) | -0.1247 |
| D(20,21,27,26) | -0.0263 | D(35,26,27,21) | 0.0267 | D(41,42,43,44) | 0.0072 |
| D(22,21,27,1) | -0.0312 | D(27,26,32,19) | 0.6264 | D(41,42,43,49) | -179.9736 |
| D(22,21,27,26) | 179.9674 | D(35,26,32,19) | -179.3672 | D(47,42,43,44) | -179.9929 |
| D(21,22,23,24) | -0.0038 | D(27,26,35,20) | -0.0175 | D(47,42,43,49) | 0.0262 |
| D(21,22,23,29) | 179.9938 | D(27,26,35,36) | 179.9743 | D(42,43,44,39) | -0.0129 |
| D(28,22,23,24) | 179.9975 | D(32,26,35,20) | 179.977 | D(42,43,44,48) | 179.989 |
| D(28,22,23,29) | -0.0049 | D(32,26,35,36) | -0.0312 | D(49,43,44,39) | 179.968 |
| D(22,23,24,25) | 0.0019 | D(1,37,38,3) | 179.8237 | D(49,43,44,48) | -0.0301 |
| D(22,23,24,30) | -179.9985 | D(1,37,38,5) | 0.1474 | D(42,43,49,50) | 149.9762 |
| D(29,23,24,25) | -179.9957 | D(2,37,38,3) | -0.1559 | D(42,43,49,51) | 29.9732 |
| D(29,23,24,30) | 0.0039 | D(2,37,38,5) | -179.8322 | D(44,43,49,50) | -30.0047 |
| D(23,24,25,20) | 0.0079 | D(44,39,40,4) | 179.9972 | D(44,43,49,51) | -150.0077 |
| D(23,24,25,31) | -179.9958 | D(44,39,40,41) | -0.0167 | D(40,39,44,43) | 0.0176 |
| D(30,24,25,20) | -179.9918 | D(45,39,40,4) | -0.0112 | D(40,39,44,48) | -179.9843 |
| D(30,24,25,31) | 0.0046 | D(45,39,40,41) | 179.9749 | D(45,39,44,43) | -179.974 |
| D(32,26,27,1) | 0.0308 | D(35,26,27,1) | -179.9747 | D(32,26,27,21) | -179.9678 |

| **Table S6.** The molecular docking result of compound 4i with the target CDK6 proteins' active pockets | | | | | |
| --- | --- | --- | --- | --- | --- |
| The target crystal structures of CDK6  PDB ID | Compound | Score Kcal/mol | Moieties from the compound | Amino acid residues | Type of interaction |
| 1XO2 | FSE | -9.2 | OH | **ASP163,** ASN150, **GLU21** | Conventional H-bond |
|  |  |  | OH | **GLY22** | C-H bond |
|  |  |  | O (Pyrone ring)  Phenyl ring | ALA162, **VAL27**  **VAL27** | Pi-Alkyl |
|  | 4i | -8.2 | C≡N | **GLU21**, GL_N_149 | Conventional H-bond |
|  |  |  | NCH_3_  C=C of pyrrole ring | ILE19, **GLU21**  **ASP163** | C-H bond |
|  |  |  | Phenyl | **VAL27**, LEU166 | Pi-Alkyl |
|  |  |  | Pyridine ring | **GLY22** | Pi-Sigma |
|  |  |  | Pyridine ring  Phenyl, pyrrole ring | LYS147  ASP145 | Pi-anion |
|  |  |  | NH of pyrrole ring | TYR24 | Unfavorable D-D |
| 2EUF | LQQ | -10.2 | NH of pyridine ring | **ILE19** | Conventional H-bond |
|  |  |  | *Pyrido*-pyrimidine ring  Cyclopentene | **LEU152, ALA41,** VAL27  VAL27 | Pi-Alkyl |
|  |  |  | Pyrido-*pyrimidine* ring  *CH_3_*CO | ILE19, LEU152  PHE98 | Pi-Sigma |
|  | 4i | -11.2 | Pyrrole ring | ASP104 | C-H bond |
|  |  |  | Indole | **ILE19,** LEU152, **ALA41**,ALA162, VAL77 | Pi-Alkyl |
|  |  |  | Phenyl  Pyrrole ring | **LEU152**  ALA162 | Pi-Sigma |
|  |  |  | Pyrrole ring | **ASP104** | Pi-anion |
| 2F2C | AP9 | -8.5 | NH | ASP163, GLN149, **ASP104** | Conventional H-bond |
|  |  |  | OH | LYS43 | C-H bond |
|  |  |  | CH_3_ | **VAL77, ALA162** | Pi-Alkyl |
|  |  |  | CH_3_  Imidazole-*pyrimidine* ring | PHE98  LEU152 | Pi-Sigma |
|  | 4i | -10.5 | NH of pyrrole ring | GLU61, ILE19 | Conventional H-bond |
|  |  |  | pyridine  Phenyl  Pyrrole | VAL27  **VAL77**  VAL27, **ALA162** | Pi-Alkyl |
|  |  |  | Phenyl | ALA162 | Pi-Sigma |
|  |  |  | Phenyl | LYS147, **ASP104** | Pi-anion |
|  |  |  | NH of pyrrole | **LYS43** | Unfavorable D-D |
| 3NUX | 3NV | -8.8 | NH of pyrazole, pyrimidine, and piprazine rings | TYR24, GLU21, **ASP104**, GLU99 | Conventional H-bond |
|  | 4i | -10.1 | C=C of pyrrole ring | ILE19 | C-H bond |
|  |  |  | N*CH_3_*  Indole  Pyrrole  Phenyl | VAL181  VAL27  ALA162  ILE19, ALA162, ALA41, LEU152 | Pi-Alkyl |
|  |  |  | Indole  Phenyl | **ASP104**  ASP163 | Pi-anion |
|  |  |  | NH of pyrrole | LYS43 | Unfavorable D-D |
| 4EZ5 | 0RS | -11.6 | CO | **LYS43** | Conventional H-bond |
|  |  |  | NH of pyridine  Tetrahydropyridine  NCH_3_ | HIS100  GLU21  **GLN149**,**ASP104** | C-H bond |
|  |  |  | Imidazole | **LYS43** | Pi-Alkyl |
|  |  |  | Imidazole  Phenyl | **VAL27**  **ILE19, LEU152** | Pi-Sigma |
|  |  |  | Imidazole  NCH_3_ | ASP163  **ASP104** | Pi-anion |
|  |  |  | NH of imidazole | LYS43 | Unfavorable D-D |
|  | 4i | -12.2 | NH of pyrrole | **ASP104** | Conventional H-bond |
|  |  |  | C=C of pyrrole ring | **GLN149** | C-H bond |
|  |  |  | N*CH_3_*  Phenyl  Pyrrole | VAL181  VAL77, ALA41  ALA162, **VAL27,** **ILE19,** **LEU152** | Pi-Alkyl |
|  |  |  | Phenyl | ALA162, **ILE19,** **LEU152** | Pi-Sigma |
|  |  |  | Phenyl | PHE98 | Pi-Pi-Stacked |
|  |  |  | NH of pyrrole | **LYS43** | Unfavorable D-D |


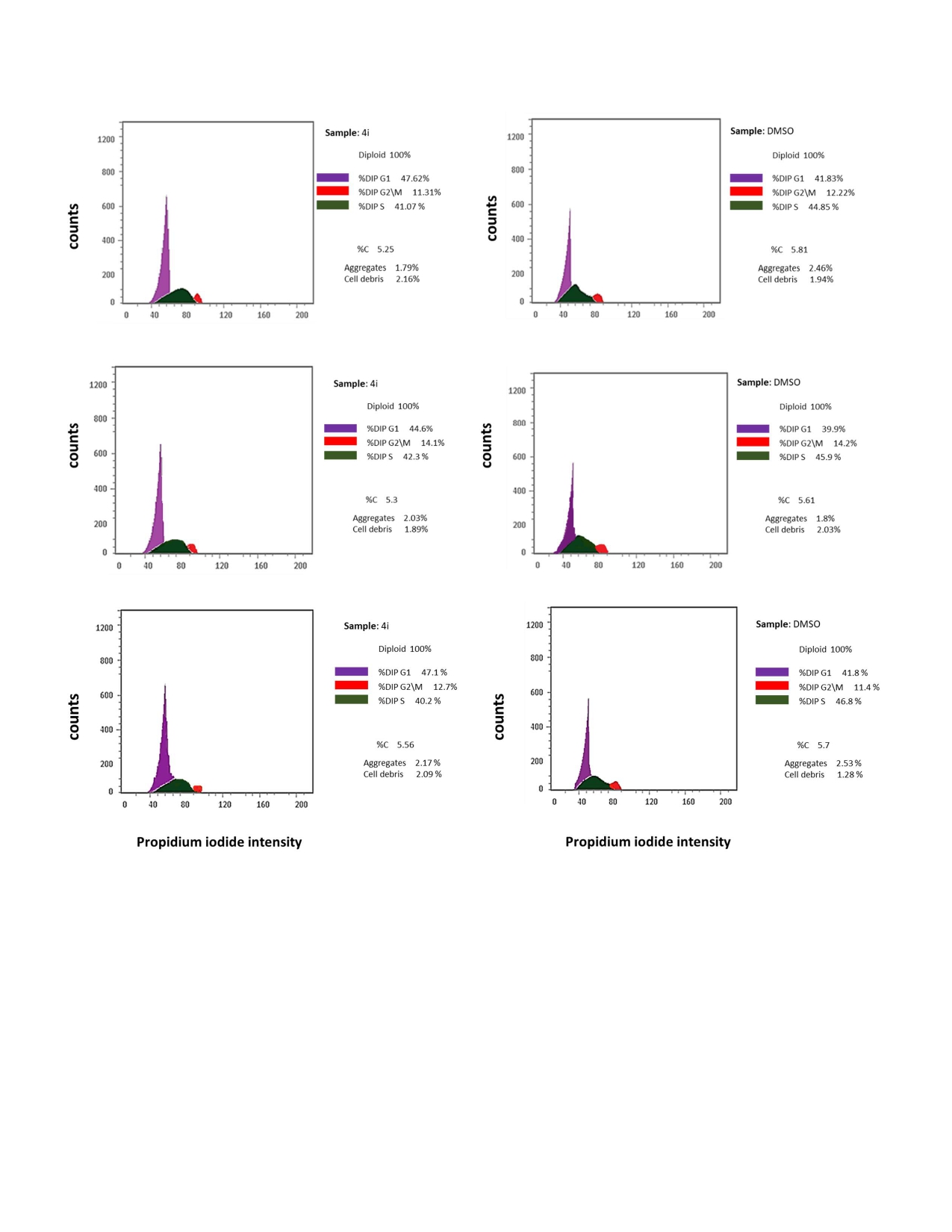


**Figure s1.** Histograms of cell cycle phases using PI staining for FACS analysis of three independent experiments


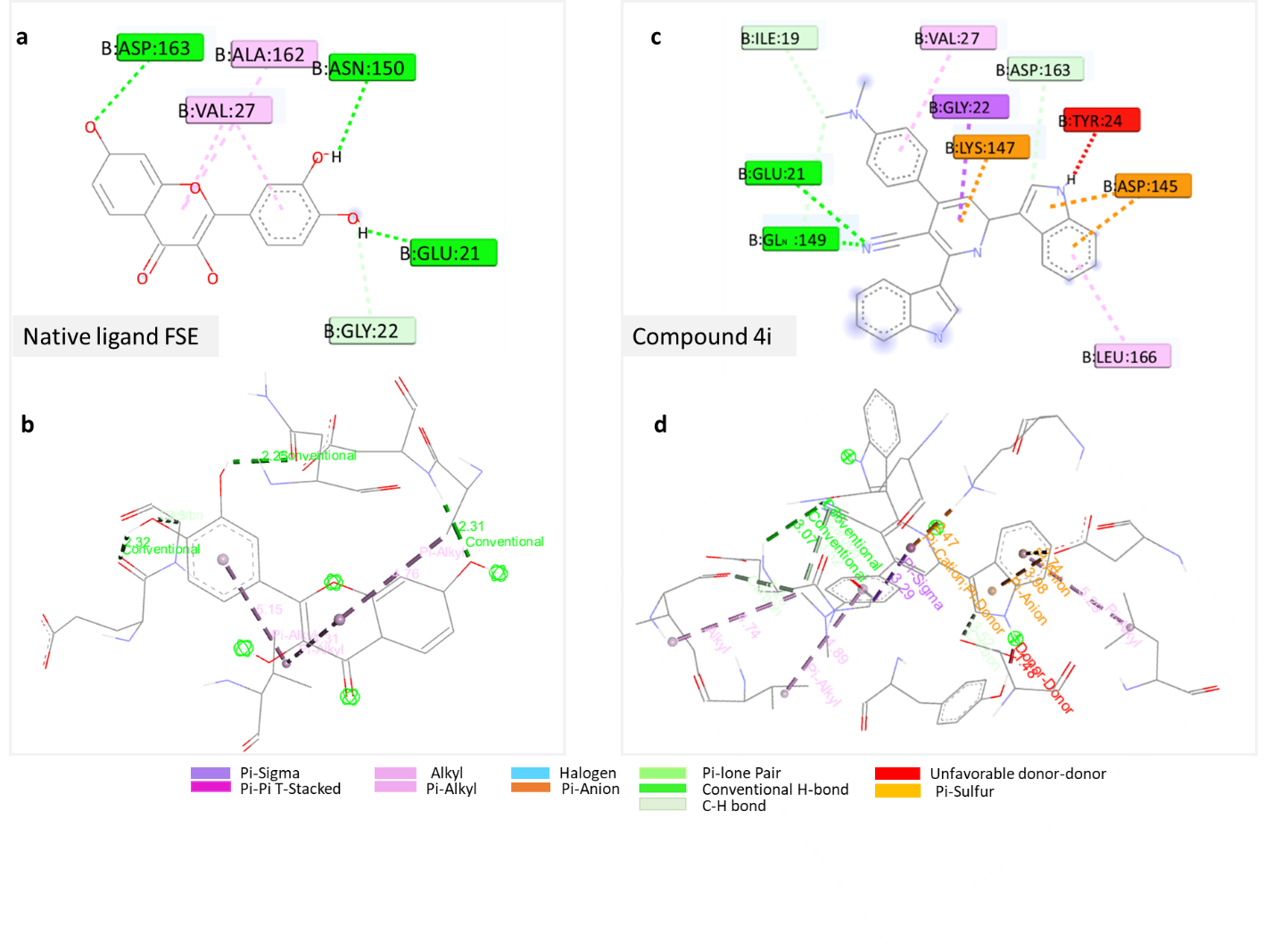


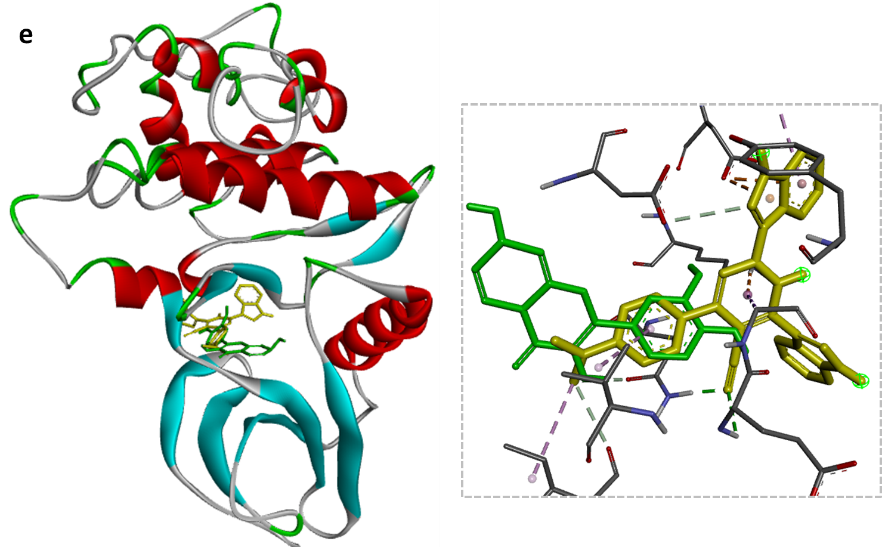


**Figure s2.** (a) The 2D interaction of native ligand FSE inside the active pocket of CDK6 (PDB:1XO2), (b) The 3D interaction of native ligand FSE inside the active pocket of CDK6 (PDB:1XO2), (c) 2D interaction of compound 4i inside the active pocket of CDK6 (PDB:1XO2), (d) 3D interaction of compound 4i inside the active pocket of CDK6 (PDB:1XO2), (e) The 3D orientation shows the laying of the re-docked ligand (green and stick) and compound 4i (yellow and stick) inside the active pocket of CDK6 (PDB:1XO2).


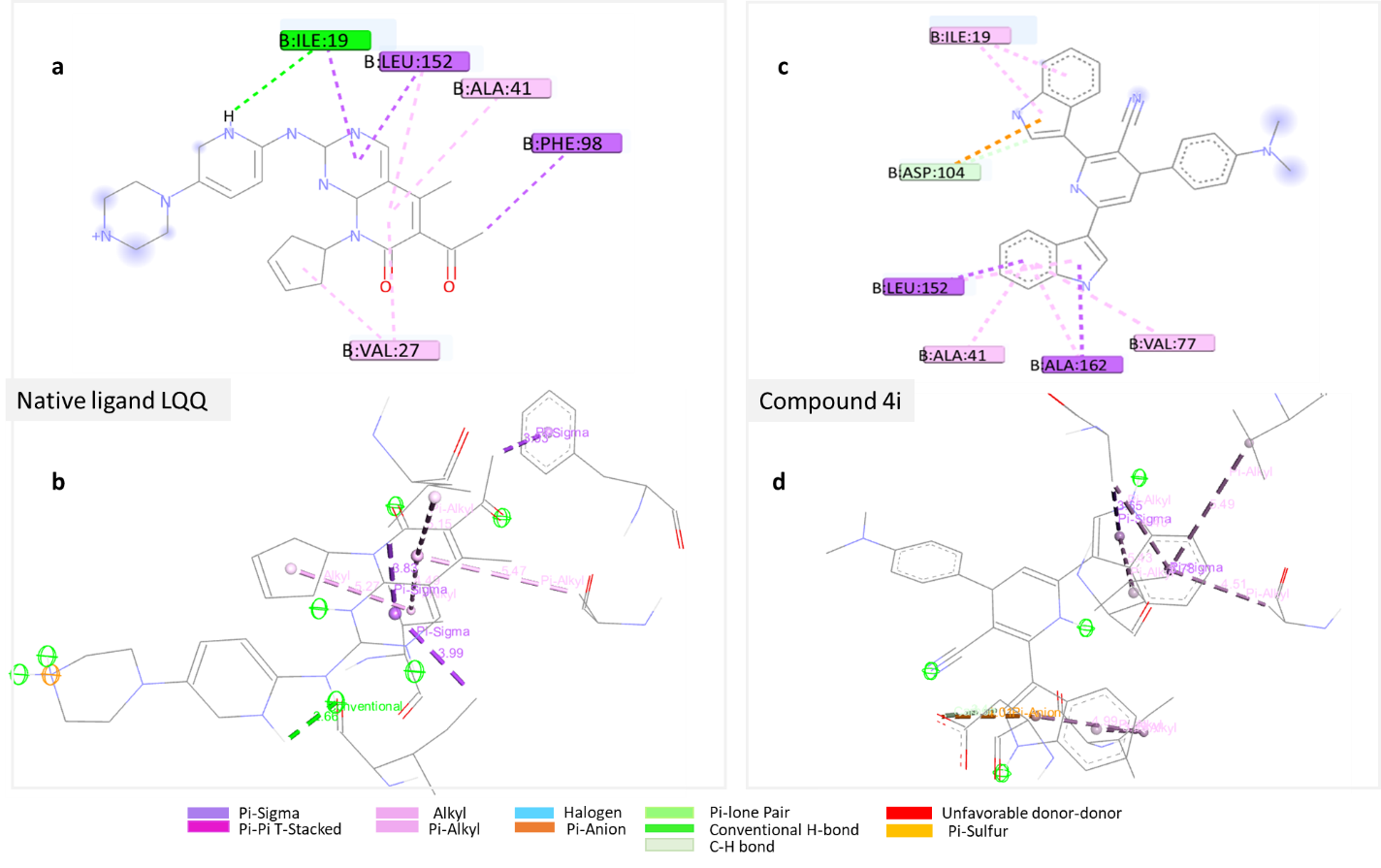


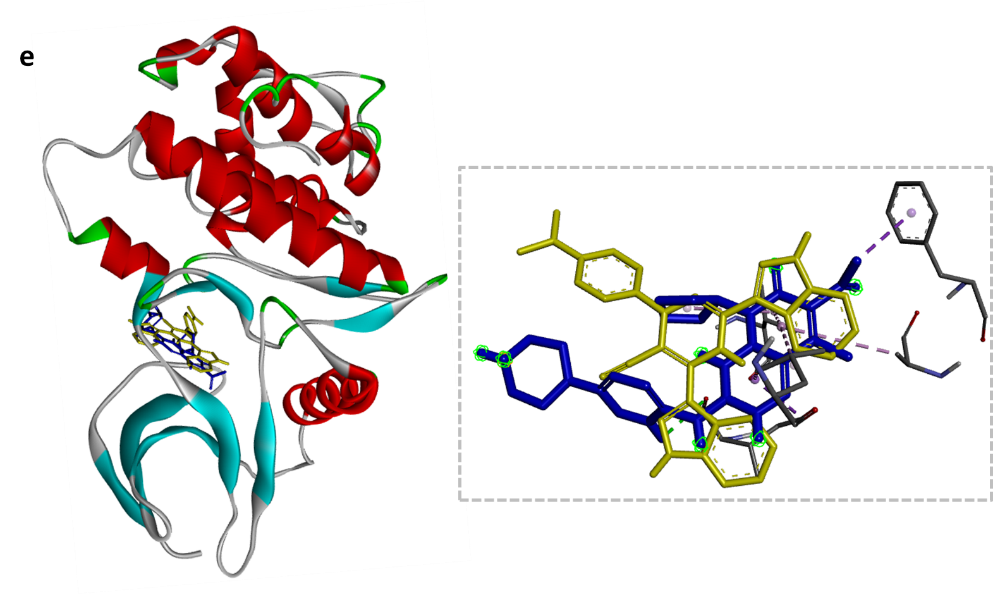


**Figure s3.** (a) The 2D interaction of native ligand LQQ inside the active pocket of CDK6 (PDB: 2EUF), (b) The 3D interaction of native ligand LQQ inside the active pocket of CDK6 (PDB: 2EUF), (c) The 2D interaction of compound 4i inside the active pocket of CDK6 (PDB: 2EUF), (d) The 3D interaction of compound 4i inside the active pocket of CDK6 (PDB: 2EUF), (e) The 3D orientation shows the laying of the re-docked ligand (LQQ, dark blue and stick) and compound (4i, yellow and stick) inside the active pocket of CDK6 (PDB: 2EUF).


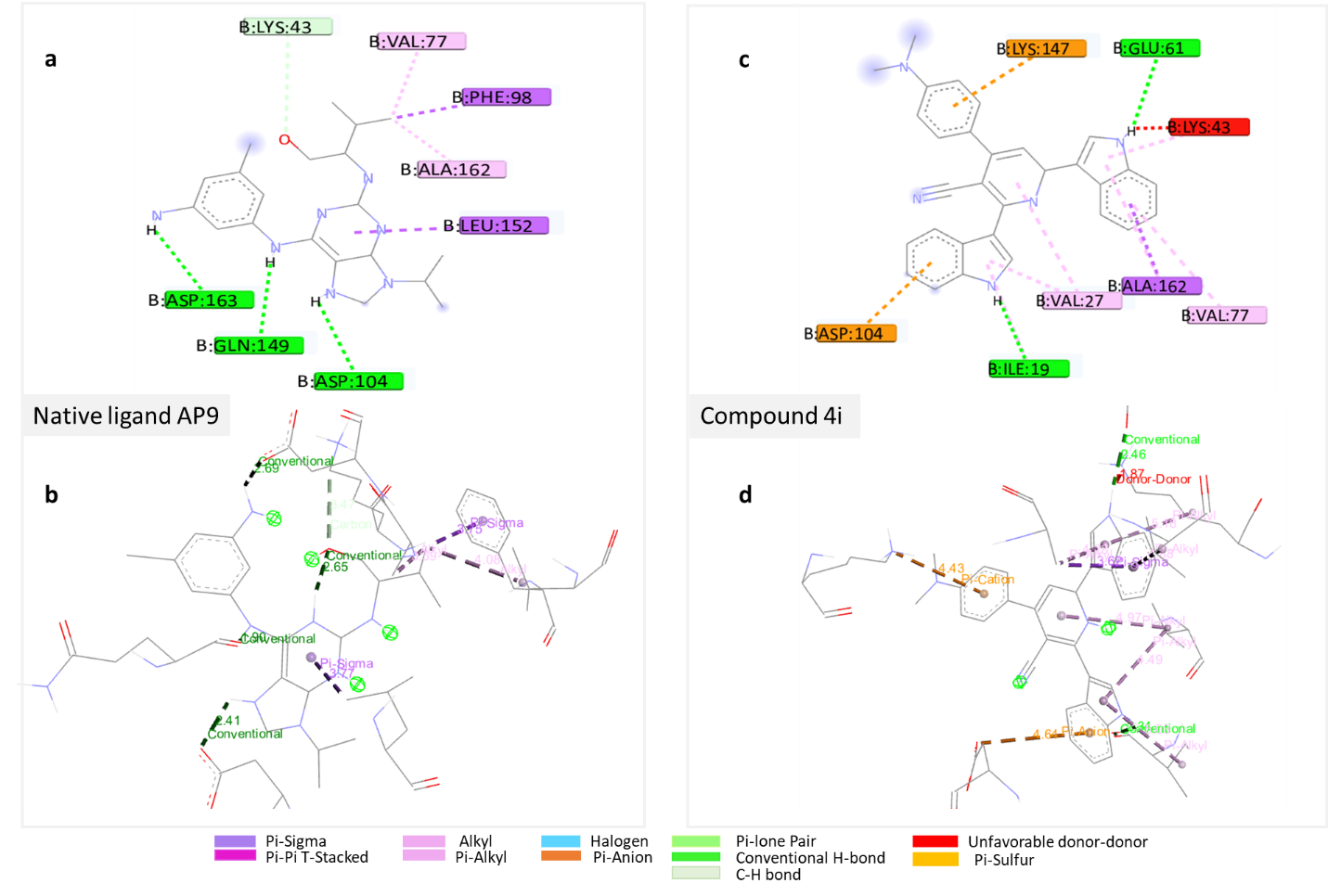


**
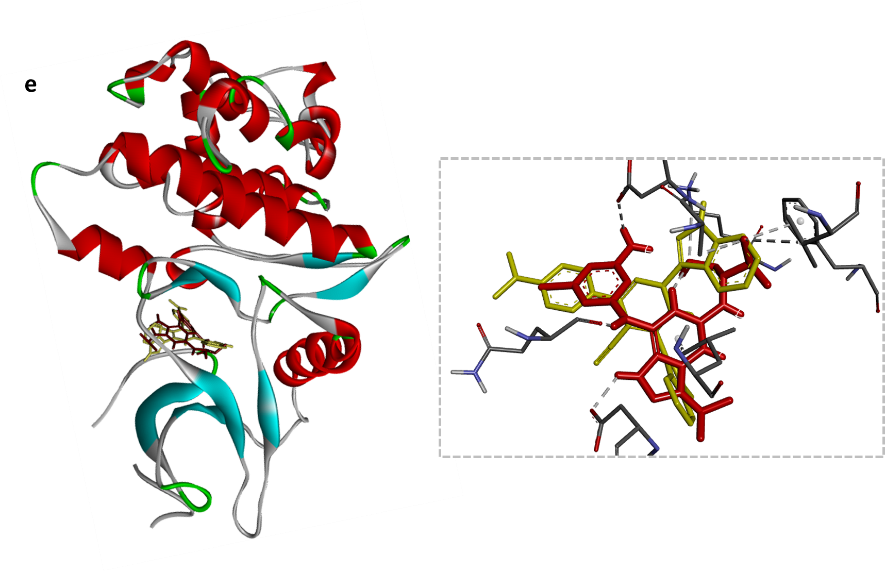
**

**Figure s4.** (a) The 2D interaction of native ligand AP9 inside the active pocket of CDK6 (PDB: 2F2C), (b) The 3D interaction of native ligand AP9 inside the active pocket of CDK6 (PDB: 2F2C), (c) The 2D interaction of compound 4i inside the active pocket of CDK6 (PDB: 2F2C), (d) The 3D interaction of compound 4i inside the active pocket of CDK6 (PDB: 2F2C), (e) The 3D orientation shows the laying of the re-docked ligand (AP9, red and stick) and compound (4i, yellow and stick) inside the active pocket of CDK6 (PDB: 2F2C).


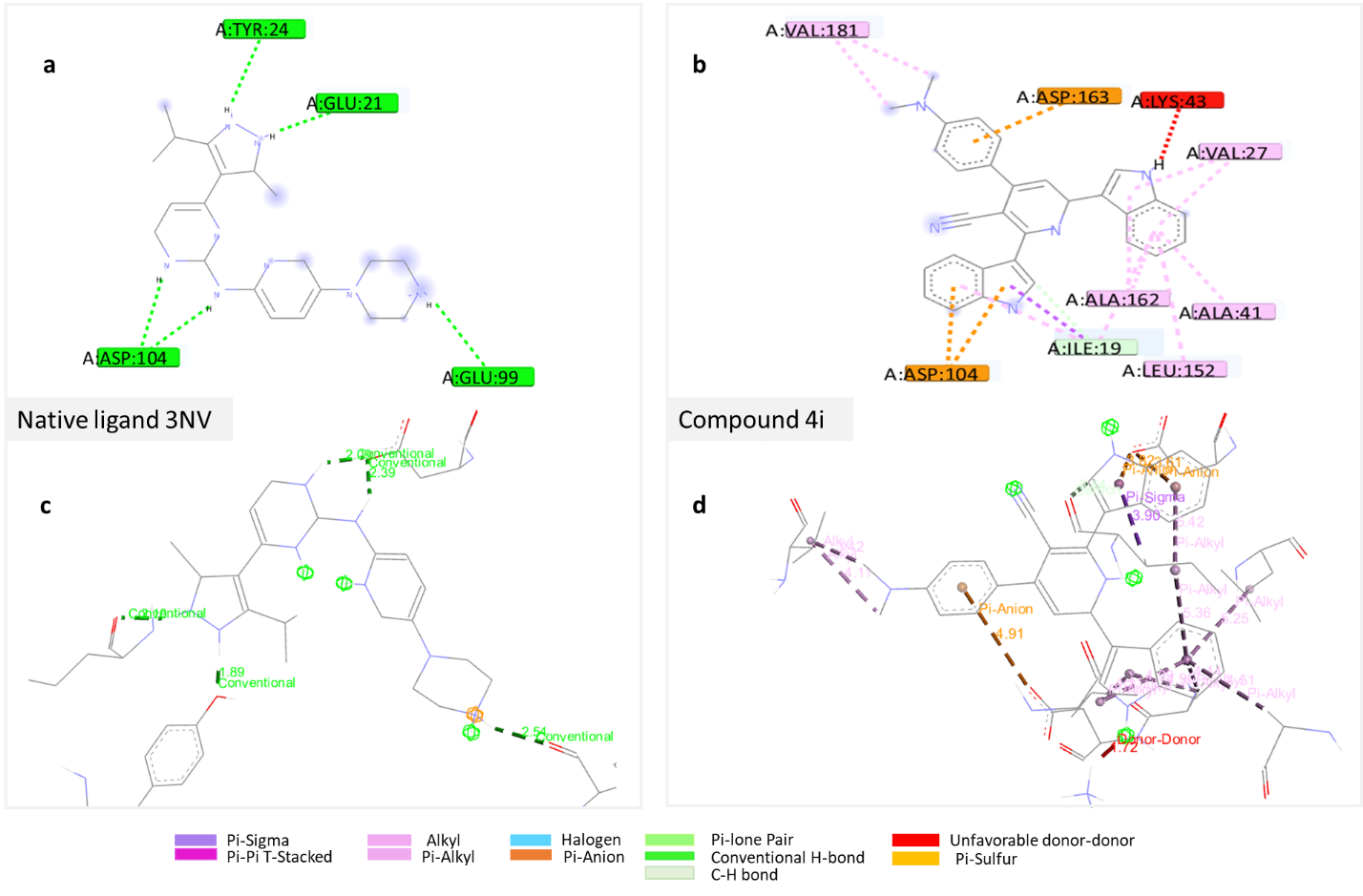


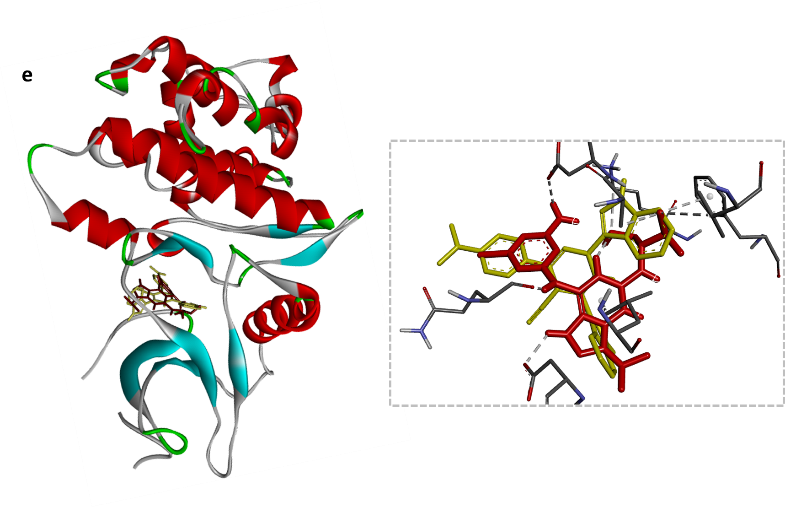


**Figure s5.** (a) The 2D interaction of native ligand 3NV inside the active pocket of CDK6 (PDB:3NUX), (b) The 3D interaction of native ligand 3NV inside the active pocket of CDK6 (PDB: 3NUX), (c) The 2D interaction of compound 4i inside the active pocket of CDK6 (PDB: 3NUX), (d) The 3D interaction of compound 4i inside the active pocket of CDK6 (PDB: 3NUX), (e) The 3D orientation shows the laying of the re-docked ligand (3NV, red and stick) and compound (4i, yellow and stick) inside the active pocket of CDK6 (PDB: 3NUX).

**
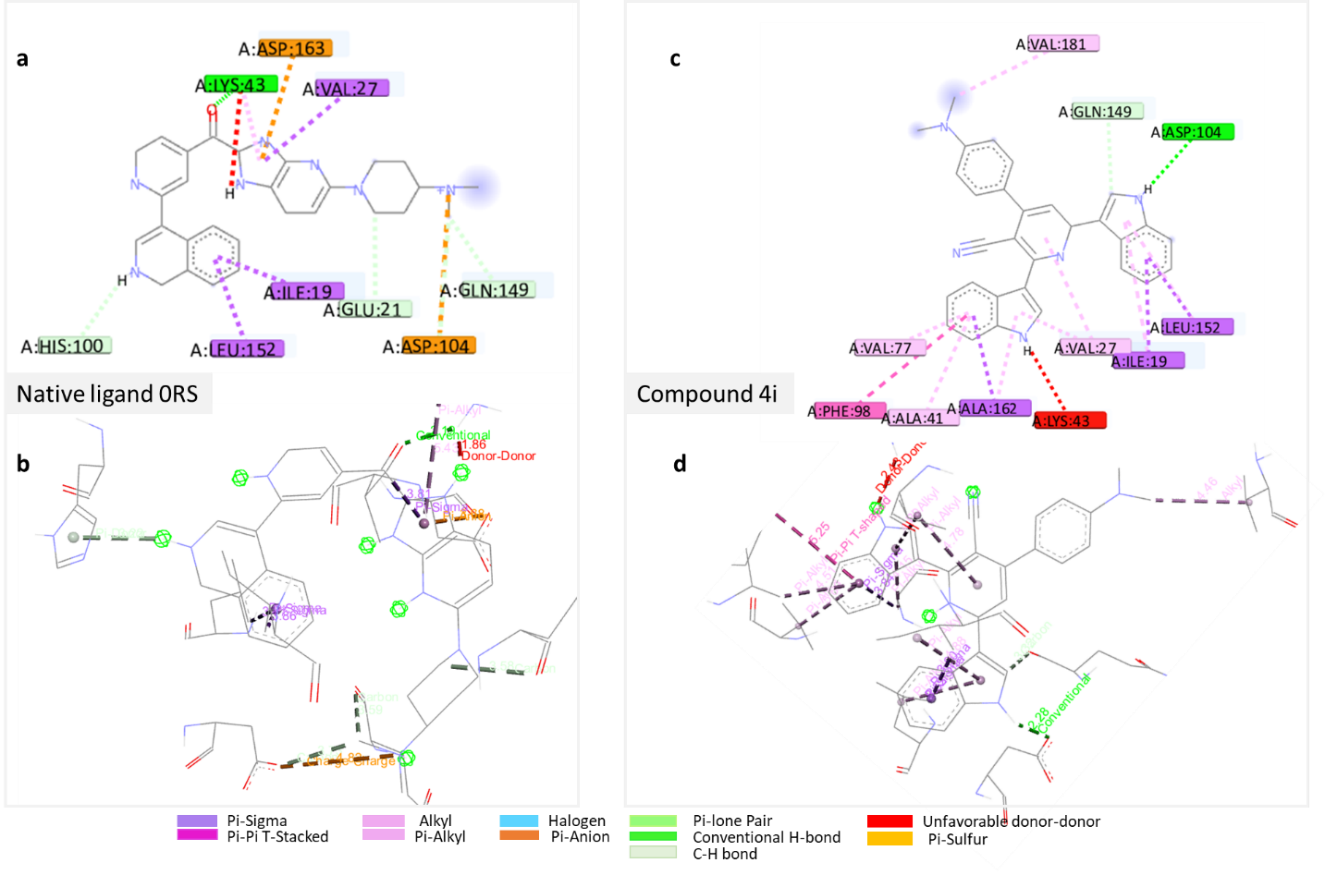
**


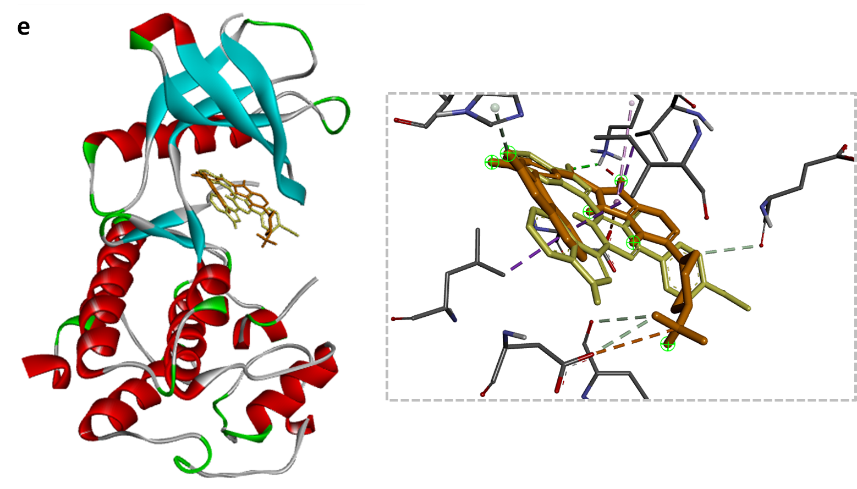


**Figure s6.** (a) The 2D interaction of native ligand 0RS inside the active pocket of CDK6 (PDB: 4EZ5), (b) The 3D interaction of native ligand 0RS inside the active pocket of CDK6 (PDB: 4EZ5), (c) The 2D interaction of compound 4i inside the active pocket of CDK6 (PDB: 4EZ5), (d) The 3D interaction of compound 4i inside the active pocket of CDK6 (PDB: 4EZ5), (e) The 3D orientation shows the laying of the re-docked ligand (0RS, brown and stick) and compound (4i, yellow and stick) inside the active pocket of CDK6 (PDB: 4EZ
